# The NIH-NIAID Schistosomiasis Resource Center at the Biomedical Research Institute: Molecular Redux

**DOI:** 10.1371/journal.pntd.0005022

**Published:** 2016-10-20

**Authors:** James J. Cody, Wannaporn Ittiprasert, André N. Miller, Lucie Henein, Margaret M. Mentink-Kane, Michael H. Hsieh

**Affiliations:** 1 Biomedical Research Institute, Rockville, Maryland, United States of America; 2 Division of Urology, Children’s National Health System, Washington, D.C., United States of America; 3 Department of Urology, The George Washington University, Washington, D.C., United States of America; Swiss Tropical and Public Health Institute, SWITZERLAND

## Abstract

Schistosomiasis remains a health burden in many parts of the world. The complex life cycle of *Schistosoma* parasites and the economic and societal conditions present in endemic areas make the prospect of eradication unlikely in the foreseeable future. Continued and vigorous research efforts must therefore be directed at this disease, particularly since only a single World Health Organization (WHO)-approved drug is available for treatment. The National Institutes of Health (NIH)–National Institute of Allergy and Infectious Diseases (NIAID) Schistosomiasis Resource Center (SRC) at the Biomedical Research Institute provides investigators with the critical raw materials needed to carry out this important research. The SRC makes available, free of charge (including international shipping costs), not only infected host organisms but also a wide array of molecular reagents derived from all life stages of each of the three main human schistosome parasites. As the field of schistosomiasis research rapidly advances, it is likely to become increasingly reliant on omics, transgenics, epigenetics, and microbiome-related research approaches. The SRC has and will continue to monitor and contribute to advances in the field in order to support these research efforts with an expanding array of molecular reagents. In addition to providing investigators with source materials, the SRC has expanded its educational mission by offering a molecular techniques training course and has recently organized an international schistosomiasis-focused meeting. This review provides an overview of the materials and services that are available at the SRC for schistosomiasis researchers, with a focus on updates that have occurred since the original overview in 2008.

## Background

Schistosomes are parasitic trematodes that cause significant liver, intestinal, and pelvic organ disease in millions of people living in tropical regions of the world. Infected snails residing in freshwater (e.g., rivers, streams, and lakes) transmit the parasites to mammalian hosts. Human activities conducted within snail-infested waters, e.g., bathing, recreation, and labor, result in schistosome exposure. Despite decades of environmental control measures as well as mass drug administration with antihelminthic drugs, schistosomiasis remains endemic in many regions of sub-Saharan Africa, Brazil, China, and the Philippines. Furthermore, a recent report describing 11 patients with *Schistosoma haematobium* infection acquired in Corsica, France, represents the first schistosomiasis cases in Europe in over 50 years [1]. Praziquantel remains the only drug that is approved by the World Health Organization (WHO) for the treatment of schistosomiasis, and therefore, the possible emergence of praziquantel-resistant *Schistosoma* species needs to be considered. All of these factors illustrate the urgent need for continued basic and applied research in schistosomiasis, including malacology, parasitology, and drug and vaccine development efforts. Fortunately, the molecular age of schistosomiasis research is well underway. Molecular tools to investigate all stages of the *Schistosoma* life cycle will be critical to usher in better diagnostics and novel antihelminthic drug and vaccine candidates.

The National Institutes of Health (NIH)–National Institute of Allergy and Infectious Diseases (NIAID)-funded Schistosome Related Reagent Repository (SR3), also known as the Schistosomiasis Resource Center (SRC) at the Biomedical Research Institute (BRI, Rockville, Maryland), offers a wide array of materials and support to investigators conducting schistosomiasis research, the extent of which those new to the field may be unaware. To enhance the visibility of the SRC, our predecessors originally highlighted the research reagents and training available through the center in 2008 [2]. Lewis et al. described the various parasite-derived products that are available for researchers, the schistosome life cycle training course, biorepository activities, and available molecular reagents. Today, the need for the SRC at BRI is keener than ever.

For instance, scientists studying schistosomiasis must make a substantial commitment to maintain the parasite’s life cycle, which necessarily involves a mammalian definitive host and the appropriate species of snail intermediate host. This is often a difficult and expensive commitment to make, particularly in the context of near-flat NIH funding and the already lean budgets allocated for tropical disease research. Beyond funding challenges, investigators often face additional problems in the allocation of sufficient lab space to this effort (especially for snail husbandry) and the limited availability of personnel with schistosome life cycle expertise. These problems can be especially daunting for the new investigator entering the field and are compounded in the case of schistosome molecular biology, since purification of sufficient amounts of parasite nucleic acids can require a large-scale parasite life cycle. Collecting parasite and vector molecular materials requires expertise, reagents, and equipment that many research groups do not have. The SRC's molecular resources are a boon to such laboratories. This is particularly important because many labs specialize in a particular aspect of schistosome biology (such as RNA regulation) and may thus have an acute need for large amounts of only one type of reagent but little to no need for other reagents.

## History, Organization, and Funding

**Fig 1:**
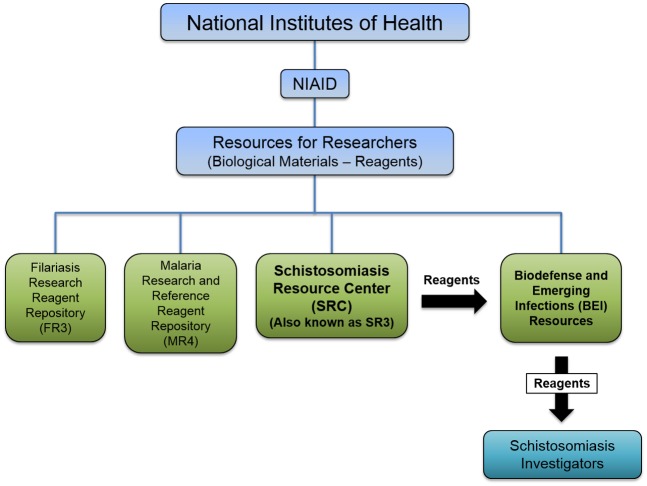
An organizational overview of the SRC at BRI. The National Institutes of Health (NIH) funds over 20 institutes and centers, including the National Institute of Allergy and Infectious Diseases (NIAID). NIAID administers a Resources for Researchers program with a subdivision dedicated to funding the production of biological materials. Several resources are supported by this mechanism, including the Schistosomiasis Resource Center (SRC). The SRC (and other resource centers, some omitted for clarity) generates research reagents that are sent to Biodefense and Emerging Infections (BEI) Resources, which was developed in 2011 to provide a web-based portal to search inventory and facilitate online ordering. Laboratories around the world interested in studying schistosomiasis are able to obtain SRC-generated reagents through BEI.

The SRC represents the core business operation of BRI, which is a 501(c) nonprofit research institute dedicated to researching neglected tropical diseases, particularly schistosomiasis. BRI houses three divisions: a biological sample repository, a research and development laboratory, and the SRC. BRI was originally organized in Illinois as the American Foundation for Biological Research in 1948 with a focus on cryobiology, before moving to Rockville, Maryland, in 1968 and assuming its current name and research focus. In 1995, BRI received its first award from NIH-NIAID to maintain schistosome life cycles for the distribution of reagents to other investigators in the field. Prior to the award of this contract to BRI, NIAID had funded a contract for schistosomiasis reagent production at other institutions for decades. The purpose of these awards was to provide investigators with a renewable and well-characterized source of standardized reagents for schistosomiasis research. The first such award was won by the University of Michigan in 1967, at which Dr. Yung-san Liang completed his training in schistosomiasis. In 1977, the contract was awarded to the University of Massachusetts (U. Mass.) at Lowell, where Dr. Liang continued his research. In parallel, BRI had an active schistosomiasis laboratory headed by Dr. Margaret Stirewalt and had been maintaining an *S*. *mansoni* life cycle to supply research reagents to investigators at NIH. Dr. Stirewalt trained a number of current leaders in schistosomiasis research, including Dr. Fred Lewis, who initially joined BRI as one of her postdoctoral fellows. Years later, Dr. Lewis took over BRI’s schistosomiasis laboratory upon the retirement of Dr. Stirewalt. Under his direction, BRI was first awarded a schistosomiasis contract in 1995, laying the foundation of the current SRC, and has successfully recompeted for the contract in 2002 and 2009 (the current award). With that initial award, Dr. Liang was recruited from U. Mass. at Lowell, bringing with him stocks of *S*. *japonicum* and *S*. *haematobium* to establish life cycles for these two species and thus expand BRI’s capabilities to include all three major human schistosomes. Dr. Lewis also recruited Dr. Mathilde (Matty) Knight, who established a molecular biology laboratory at BRI. In the course of her research, Dr. Knight initially provided select molecular reagents for other schistosomiasis investigators upon request using surplus life cycle materials. However, she recognized that the need existed for a centralized source of well-characterized molecular reagents (of known concentration, purity, restriction enzyme patterns, etc.) and that this would be a logical extension of BRI’s existing life cycle production work. With this goal in mind, the contract was amended in 2010 to include funding for production of molecular reagents, and thus, the molecular reagent production facility was consolidated with the existing life cycle facility then known as the Schistosome Related Reagent Repository (SR3). Items were initially distributed directly from the SR3 before the formation of the current partnership with the Biodefense and Emerging Infections Resources program (BEI Resources, http://www.beiresources.org/) in 2011. The NIH established BEI as a central processing entity that allows laboratories to request materials from several NIH-funded resource centers, including those for schistosomiasis and malaria research (Fig 1). In 2014, the SR3 was rebranded as the Schistosomiasis Resource Center (SRC) to reflect the fact that BEI now served as the repository of molecular reagents and to more accurately encompass the SRC’s additional activities in education and outreach.

BRI is presently managed by an executive director, Dr. Paul Nisson, and a board of directors comprised of external scientists and administrative advisors. In 2011, BRI established a scientific advisory committee that convenes two times per year to oversee and advise the SRC on scientific matters. The SRC is currently directed by Dr. Michael Hsieh, who has served as principal investigator since 2014, and Dr. Margaret Mentink-Kane, who has managed production of life cycle and molecular reagents since 2015. Contact information for these and other key personnel can be found on the BRI website: www.afbr-bri.com/schistosomiasis/contacts. Financial support of the SRC is provided by the “Maintenance, Development and Production of Schistosomiasis Parasites, Reagents and Assays” contract administered by the Parasitology and International Programs Branch (PIPB) of the Division of Microbiology and Infectious Diseases of NIAID (Fig 1). This award supports both the life cycle and molecular work of the SRC.

## Available Schistosomiasis Research Materials

**Fig 2:**
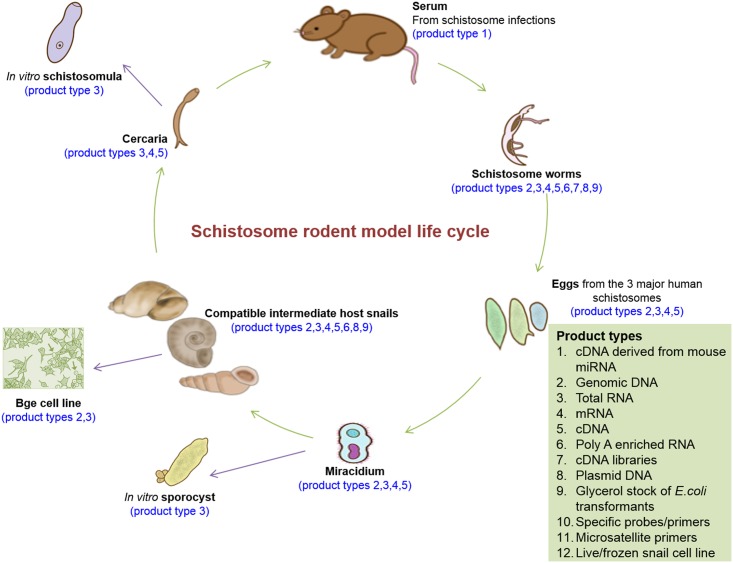
Schematic of molecular materials available from BEI Resources. A variety of molecular materials derived from all life stages of three *Schistosoma* species (*S*. *mansoni*, *S*. *haematobium*, and *S*. *japonicum*) and infected host species are available as indicated. Items 10, 11, and 12 are future products.

Many of the schistosome-specific molecular tools can be obtained free of charge by scientists through the SRC. This benefit was made possible because, as detailed above, long before the molecular era of schistosomiasis research, the NIH-NIAID had the foresight to establish a resource from which investigators could obtain various schistosome life stages without having to expend the effort and funds necessary to maintain *Schistosoma* spp. life cycles on their own. Since 2003, the SRC has served as a central facility for the collection and long-term storage of schistosome and host molecular reagents. At the time of the Lewis et al. publication in 2008, the SRC molecular product collection, assembled with critical guidance by Dr. Knight, consisted of complementary DNA (cDNA) libraries for *S*. *mansoni* and a few reagents (primarily cDNAs) for *Biomphalaria glabrata*. It was anticipated at the time that an expanded range of reagents (cDNA libraries, primers, plasmid DNAs, etc.) would become available for additional species, and this goal has been fulfilled (Fig 2). These reagents have been duplicated and deposited at BEI for distribution, free of charge, to the scientific community.

**Fig 3:**
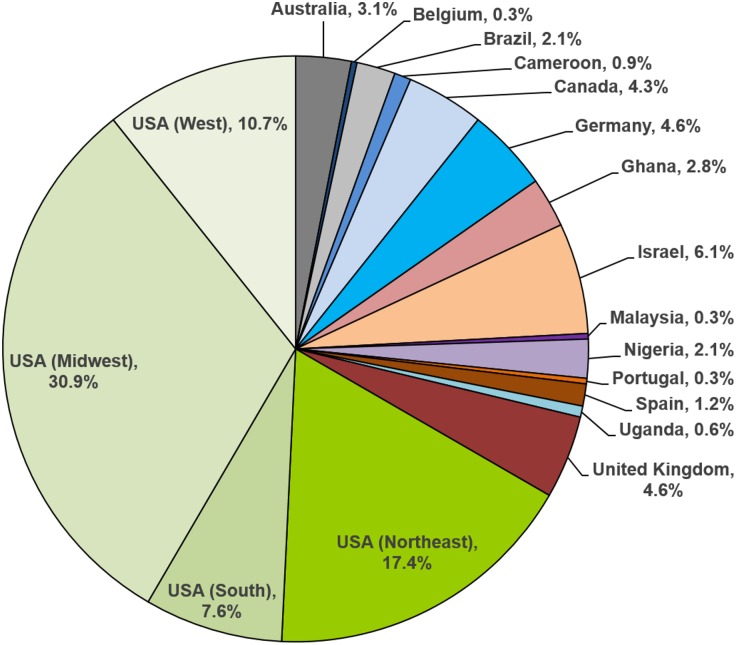
The geographic locations of requests for SRC-generated molecular reagents from 2011–2016. Requests for SRC-generated molecular reagents have come from 15 countries over the years 2011 through July 2016. Shown is the percentage of total requests attributable to each country. The United States as a whole accounts for 66.7% of all items ordered, distributed as shown among the Northeast (Washington, D.C., Massachusetts, Maryland, New York, and Pennsylvania), South (Alabama, Georgia, Texas, and Virginia), Midwest (Iowa, Illinois, Indiana, Michigan, Minnesota, Ohio, and Wisconsin), and West (California, Colorado, New Mexico, and Utah).

As a centralized resource, the NIH-NIAID SRC translated, and continues to translate, into cost savings for both NIH-NIAID and principal investigators by freeing up staff and infrastructure costs on grants and allowing investigators to allocate more funds to targeted research goals. Since October of 2010, the SRC has offered molecular reagents free of cost to any laboratory. Currently, the SRC has an inventory of over 180 user-ready molecular products, including total RNA, messenger RNA, enriched poly A mRNA, and cDNA, derived from adult schistosomes, eggs, miracidia, and cercariae of the three major human schistosome parasites: *S*. *haematobium*, *S*. *japonicum*, and *S*. *mansoni*, as well as total RNA from *S*. *mansoni* (Naval Medical Research Institute [NMRI] strain) in vitro*-*transformed sporocysts and schistosomula. The SRC also offers a large selection of molecular products from *Schistosoma* intermediate hosts (*Bulinus* spp., *Oncomelania* spp., and *Biomphalaria* spp.) and a *B*. *glabrata* embryonic cell line (designated Bge) that is currently the only mollusk cell line suitable for long-term culture and genetic manipulation. The SRC has recently expanded its offerings to include an *S*. *haematobium* (Egyptian strain) adult worm cDNA library and plasmid vectors containing selected *S*. *haematobium* genes, as well as cDNA synthesized from microRNAs (miRNAs) isolated from the plasma of infected rodent hosts (*S*. *haematobium*, *S*. *japonicum*, and *S*. *mansoni* infections) (Fig 2). Since 2011, over 300 requests for SRC molecular reagents have been made, originating from 15 countries (Fig 3). In terms of molecular reagents, the most frequently requested catalog items have included total RNA and genomic DNA derived from adult *S*. *mansoni* (NMRI), genomic DNA from adult *S*. *haematobium*, and total RNA from *S*. *mansoni* (NMRI) cercaria and eggs. For life cycle reagents, the highest-demand items continue to be *S*. *mansoni* (NMRI)-exposed *B*. *glabrata* snails and mice, as well as unexposed *B*. *glabrata* (NMRI) snails. Also frequently requested are *Bulinus truncatus* snails exposed to *S*. *haematobium* and *B*. *glabrata* snails exposed to *S*. *mansoni* (PR-1 strain).

## How to Apply for Materials

Because of the importance of the SRC’s mission, investigators at an array of institutions and organizations are eligible to receive materials: academic, nonprofit, industry, and government (local and national, worldwide). Investigators wishing to obtain SRC-generated schistosomiasis research reagents are referred to the BEI website (www.beiresources.org/). Here, prospective users must first register as a Biosafety Level 1 or 2 registrant, depending upon the materials they intend to use. BEI provides forms that can be downloaded directly from the website (S1 File). These forms allow the registrant to submit the following information: Registrant Application, Material Transfer Agreement, Biographical Sketch, and Biosafety Level 2 checklist for the facility that will be receiving the material. Upon receipt of all required forms, BEI will inform the registrant that his or her application is complete, and she or he will have the opportunity to create a profile for logging in to the website. The new user can then refer to the BEI catalog for a complete listing of all available reagents, each of which are assigned a catalog number. Available items can be browsed by resource/disease area, depositor (such as the SR3), or organism. Users can also search by specific catalog number (if known) or key words. Each item has a product sheet (describing the material, its origin, and the methods used for its production) and a certificate of analysis with lot-specific information such as concentration and purity. The user can then add an item or items of interest to his or her online cart, review the order, and submit it as with any typical vendor.

To properly attribute the valuable funding that allows the SRC to fulfill its mission, NIAID asks that users acknowledge this fact in any publication that results from SRC-generated materials. To that end, materials should be acknowledged as such: “[reagent provided: e.g., *B*. *glabrata* snails] provided by the NIAID Schistosomiasis Resource Center of the Biomedical Research Institute (Rockville, MD) through NIH-NIAID contract HHSN2722010000051 for distribution through BEI Resources.”

## Complementary Resources

To promote schistosomiasis research, we wish to direct the reader to molecular resources outside of our center that are nonetheless valuable and potentially complementary to SRC reagents. A comprehensive resource for genomic information on a wide variety of nematode and platyhelminth species, including seven *Schistosoma* species, can be found at WormBase ParaSite (http://parasite.wormbase.org/). An updated version of the *Schistosoma* genome database (SchistoDB) is available via http://SchistoDB.net. This integrated website provides the latest genomic sequence and annotation data for *S*. *haematobium*, *S*. *japonicum*, and *S*. *mansoni* [3]. Since the publication of our original SRC overview in 2008, the ensuing years have seen the publication of the genomes for all three major human schistosome species [4–6], with additional genomic and transcriptomic data for *S*. *mansoni* in particular [7]. The subsequent publication of a genomic linkage map for *S*. *mansoni*, the first for a helminth species [8], has allowed for further research probing the basis of drug (oxamniquine) resistance by classical linkage mapping techniques [9] and by more efficient methods such as exome capture and extreme quantitative trait locus analysis [10]. Host tissue transcriptomes have been characterized for the mouse bladder following *S*. *haematobium* egg exposure as well as for the liver and spleen following *S*. *japonicum* infection [11–15]. Next-generation sequencing (NGS) has been utilized to identify genes under selective pressure in *S*. *mansoni* and has aided in the search for new molluscicide formulations against *Oncomelania hupensis* [16,17]. Furthermore, since the first report of particle bombardment to transfect adult worms [18], other groups have continued to advance schistosome transgenics: in 2012, Rinaldi et al. described germline transmission of a transgene introduced into parasite eggs via murine leukemia virus [19,20]. The understanding of epigenetic regulation in lower organisms is advancing as molecular tools become available to examine invertebrate epigenetics including *B*. *glabrata* and *Schistosoma* [21,22]. Clinical studies have shown that modification of the host genome occurs following infection with *S*. *haematobium* [23–25], and deeper studies are much needed in this area. Similarly, the influence of the microbiome (both host and parasite) on schistosomiasis infection is only beginning to be explored. Recent studies have demonstrated that infection with the liver fluke *Opisthorchis viverrini* alters the makeup and distribution of the microbiome in an animal model [26] and that differences exist in the fecal microbiome of humans infected versus uninfected with *S*. *haematobium* [27]. Another recent report characterized the microbiome of *B*. *glabrata* [28]. Thus, schistosome-related omics research is thriving, which will drive continued and expanded need for SRC reagents.

## Education and Outreach

In addition to supplying a variety of reagents to the community, in 2012 the SRC began offering a hands-on annual course to introduce students, postdoctoral fellows, and investigators to fundamental molecular techniques that are useful for schistosomiasis research. This course is offered in addition to the ongoing training courses dedicated to the schistosome life cycle and thus represents an expansion of the SRC’s educational mission. For both courses, enrollment is capped (at 5–6 for the molecular course or up to 10 for the life cycle) in order to maximize the ability of students to interact closely with SRC staff and invited speakers. Applications are not required, but attendees must notify the SRC of their intent to attend so that a position can be reserved. Courses are typically organized in the spring (life cycle) and fall (life cycle and molecular), with the exact dates being announced 2–3 months prior on the BRI website. Announcements are also distributed directly to the SRC client list via email. For both courses, the rosters have included a mix of individuals at all academic levels: graduate students, technicians, postdoctoral fellows, scientists, and senior investigators. Sample course outlines for both the life cycle and the molecular course are included as a supplemental file (S2 File). The new molecular course is offered free of charge to the research community and features important techniques such as gene knockdown in snails and schistosome parasites by double-stranded RNA (dsRNA) (synthesized in-house) and miRNA. The course has also taught attendees polyethylenimine-based techniques for transfection of target cells and organisms with dsRNA, reporter miRNA, and plasmid vectors. This topic in particular illustrates the immediate success of the molecular course. Attendees from the 2012 course, Shuang Liang and Emmitt Jolly, published a manuscript based on the techniques developed in the class [29]. While some general techniques have recurred in multiple courses, the overall focus varies from year to year as the SRC seeks to offer cutting-edge topics that track the current interests of the field. In 2015, the course theme was the epigenetics of the intermediate host snail. These courses are valuable beyond generating publications, since insights are shared regarding which molecular reagents work best with invertebrate starting material. Lectures are given by SRC staff and invited external speakers and cover a range of relevant topics, including miRNA gene regulation mechanisms, DNA methylation, and schistosome-specific bioinformatics. Hands-on laboratory exercises have involved nucleic acid purification and quality assurance, cDNA synthesis, gene transfection, gene transcription quantification, and measurement of DNA methylation in schistosome parasites and/or snails. Finally, the SRC-BRI website offers a comprehensive array of standard operating procedures for parasite and host species maintenance and molecular protocols that are a valuable resource for the schistosomiasis research community (www.afbr-bri.com/schistosomiasis/standard-operating-procedures/).

## The Symposium for International Research and Innovations in Schistosomiasis

The SRC will continue to track and contribute to developments in the field of schistosomiasis and in infectious disease research in general, in order to meet the changing needs of the research community and expand the scope of the reagents offered, with a recent emphasis on molecular reagents. In fact, the SRC at BRI has taken a leading role in fostering advances in schistosomiasis research. In 2016, BRI and the George Washington University (Washington, D.C.) collaborated to sponsor an international meeting intended as one of the first of its kind that will focus solely on schistosomiasis research: the Symposium for International Research and Innovations in Schistosomiasis (SIRIS). The 2016 meeting, “Defining the Global Impact of Schistosomiasis,” brought together 70 attendees. Experts from a variety of academic, industry, and private organizations presented their latest findings, ranging from an epidemiological level to a molecular level, on topics including the effectiveness of current therapeutic strategies, mass drug administration efforts, parasite biology, vaccines, omics, and disease pathology. Given the overwhelmingly positive feedback gathered from attendees, this event promises to become a key forum for the exchange of ideas in the schistosomiasis research community for years to come. Through efforts such as these, the SRC at BRI will continue to fulfill its mission of enabling the research community to combat schistosomiasis.

**Key Learning Points**

- Schistosomiasis remains a major health burden in many parts of the world, and continued research efforts are critical.
- The SRC provides investigators with a wide variety of parasite- and host-derived raw materials and has fulfilled over 300 requests since 2011.
- The SRC partnered with BEI in 2011 to streamline the inventory and shipping of the more than 180 products now available.
- Rapid advances in schistosomiasis research, such as the recently completed SchistoDB genome database, point to an increasingly molecular focus for the field.
- The SRC has instituted a molecular techniques training course to complement its existing life cycle course, now offers numerous molecular reagents, and recently hosted an international schistosomiasis-focused scientific meeting.

**Top Five Papers in the Field**

1. Young ND, Jex AR, Li B, Liu S, Yang L, Xiong Z, et al. Whole-genome sequence of *Schistosoma haematobium*. Nat Genet. 2012 Jan 15;44(2):221–5. doi:10.1038/ng.1065.
2. Zhou Y, Zheng H, Chen X, Zhang L, Wang K, Guo J, et al. The *Schistosoma japonicum* genome reveals features of host-parasite interplay. Nature. 2009 Jul 16; 460(7253):345–351. doi:10.1038/nature08140.
3. Berriman M, Haas BJ, LoVerde PT, Wilson RA, Dillon GP, Cergueira GC, et al. The genome of the blood fluke *Schistosoma mansoni*. Nature. 2009 Jul 16;460(7253):352–8. doi:10.1038/nature08160.
4. Rinaldi G, Eckert SE, Tsai IJ, Suttiprapa S, Kines KJ, Tort JF, et al. Germline transgenesis and insertional mutagenesis in *Schistosoma mansoni* mediated by murine leukemia virus. PLoS Pathog. 2012;8:16. doi:10.1371/journal.ppat.1002820.
5. Cai P, Gobert GN, You H, McManus DP. The Tao survivorship of schistosomes: Implications for schistosomiasis control. Int J Parasitol. 2016 Feb 9. pii: S0020-7519(16)00023-0. doi: 10.1016/j.ijpara.2016.01.002.

## Supporting Information

S1 File BEI registration forms. Investigators wishing to use SRC-generated life cycle and molecular reagents must first register on the BEI website. Included are blank registration forms for Biosafety Level 1 and 2 laboratories.
(PDF)

S2 File Course outlines for the SRC’s life cycle and molecular training courses. The SRC at BRI offers a life cycle course twice yearly and an annual molecular skills course. Included are example outlines for each course. While the life cycle course features recurring but continually refined content, the molecular course topic is changed yearly to track updates in the field.
(PDF)

## References

[1] Holtfreter MC, Moné H, Müller-Stöver I, Mouahid G, Richter J. Schistosoma haematobium infections acquired in Corsica, France, August 2013. Eurosurveillance. 2014;19 10.2807/1560-7917.ES2014.19.22.20821 24925456

[2] Lewis FA, Liang YS, Raghavan N, Knight M. The NIH-NIAID schistosomiasis resource center. PLoS Negl Trop Dis. 2008 10.1371/journal.pntd.0000267 18665228 PMC2480520

[3] Zerlotini A, Aguiar ERGR, Yu F, Xu H, Li Y, Young ND, et al SchistoDB: An updated genome resource for the three key schistosomes of humans. Nucleic Acids Res. 2013;41 10.1093/nar/gks1087 23161692 PMC3531198

[4] Zhou Y, Zheng H, Chen Y, Zhang L, Wang K, Guo J, et al The Schistosoma japonicum genome reveals features of host-parasite interplay. Nature. 2009;460: 345–351. 10.1038/nature08140 19606140 PMC3747554

[5] Berriman M, Haas BJ, LoVerde PT, Wilson RA, Dillon GP, Cerqueira GC, et al The genome of the blood fluke Schistosoma mansoni. Nature. 2009;460: 352–8. 10.1038/nature08160 19606141 PMC2756445

[6] Young ND, Jex AR, Li B, Liu S, Yang L, Xiong Z, et al Whole-genome sequence of Schistosoma haematobium. Nat Genet. 2012;44: 221–225. 10.1038/ng.1065 22246508

[7] Protasio A V., Tsai IJ, Babbage A, Nichol S, Hunt M, Aslett MA, et al A systematically improved high quality genome and transcriptome of the human blood fluke Schistosoma mansoni. PLoS Negl Trop Dis. 2012;6 10.1371/journal.pntd.0001455 22253936 PMC3254664

[8] Criscione CD, Valentim CLLL, Hirai H, LoVerde PT, Anderson TJCC. Genomic linkage map of the human blood fluke Schistosoma mansoni. Genome Biol. 2009;10: R71 10.1186/gb-2009-10-6-r71 19566921 PMC2718505

[9] Valentim CLL, Cioli D, Chevalier FD, Cao X, Taylor AB, Holloway SP, et al Genetic and molecular basis of drug resistance and species-specific drug action in schistosome parasites. Science. 2013;342: 1385–9. 10.1126/science.1243106 24263136 PMC4136436

[10] Chevalier FD, Valentim CL, LoVerde PT, Anderson TJ. Efficient linkage mapping using exome capture and extreme QTL in schistosome parasites. BMC Genomics. 2014;15: 617 10.1186/1471-2164-15-617 25048426 PMC4117968

[11] Ray D, Nelson TA, Fu CL, Patel S, Gong DN, Odegaard JI, et al Transcriptional Profiling of the Bladder in Urogenital Schistosomiasis Reveals Pathways of Inflammatory Fibrosis and Urothelial Compromise. PLoS Negl Trop Dis. 2012;6 10.1371/journal.pntd.0001912 23209855 PMC3510078

[12] Burke ML, McManus DP, Ramm GA, Duke M, Li Y, Jones MK, et al Co-ordinated gene expression in the liver and spleen during schistosoma japonicum infection regulates cell migration. PLoS Negl Trop Dis. 2010;4 10.1371/journal.pntd.0000686 20502518 PMC2872641

[13] Burke ML, McManus DP, Ramm GA, Duke M, Li Y, Jones MK, et al Temporal expression of chemokines dictates the hepatic inflammatory infiltrate in a murine model of schistosomiasis. PLoS Negl Trop Dis. 2010;4 10.1371/journal.pntd.0000598 20161726 PMC2817718

[14] Chuah C, Jones MK, Burke ML, Owen HC, Anthony BJ, McManus DP, et al Spatial and temporal transcriptomics of Schistosoma japonicum-induced hepatic granuloma formation reveals novel roles for neutrophils. J Leukoc Biol. 2013;94: 1–13. 10.1189/jlb.1212653 23709687

[15] Perry CR, Burke ML, Stenzel DJ, McManus DP, Ramm GA, Gobert GN. Differential expression of chemokine and matrix re-modelling genes is associated with contrasting schistosome-induced hepatopathology in murine models. PLoS Negl Trop Dis. 2011;5 10.1371/journal.pntd.0001178 21666794 PMC3110159

[16] Clément JAJ, Toulza E, Gautier M, Parrinello H, Roquis D, Boissier J, et al Private Selective Sweeps Identified from Next-Generation Pool-Sequencing Reveal Convergent Pathways under Selection in Two Inbred Schistosoma mansoni Strains. PLoS Negl Trop Dis. 2013;7 10.1371/journal.pntd.0002591 24349597 PMC3861164

[17] Zhao QP, Xiong T, Xu XJ, Jiang M Sen, Dong HF. De novo transcriptome analysis of oncomelania hupensis after molluscicide treatment by next-generation sequencing: Implications for biology and future snail interventions. PLoS ONE. 2015;10 10.1371/journal.pone.0118673 25775015 PMC4361594

[18] Davis RE, Parra A, LoVerde PT, Ribeiro E, Glorioso G, Hodgson S. Transient expression of DNA and RNA in parasitic helminths by using particle bombardment. Proc Natl Acad Sci U S A. 1999;96: 8687–92. http://www.pubmedcentral.nih.gov/articlerender.fcgi?artid=17577&tool=pmcentrez&rendertype=abstract 10.1073/pnas.96.15.8687 10411936 PMC17577

[19] Rinaldi G, Eckert SE, Tsai IJ, Suttiprapa S, Kines KJ, Tort JF, et al Germline transgenesis and insertional mutagenesis in Schistosoma mansoni mediated by murine leukemia virus. PLoS Pathog. 2012;8: 16 10.1371/journal.ppat.1002820 22911241 PMC3406096

[20] Hagen J, Scheerlinck JPY, Young ND, Gasser RB, Kalinna BH. Prospects for vector-based gene silencing to explore immunobiological features of schistosoma mansoni. Adv Parasitol. 2015;88: 85–122. 10.1016/bs.apar.2015.02.002 25911366

[21] Fneich S, Dheilly N, Adema C, Rognon A, Reichelt M, Bulla J, et al 5-methyl-cytosine and 5-hydroxy-methyl-cytosine in the genome of Biomphalaria glabrata, a snail intermediate host of Schistosoma mansoni. Parasit Vectors. 2013;6: 167 10.1186/1756-3305-6-167 23742053 PMC3681652

[22] Ittiprasert W, Miller A, Knight M, Tucker M, Hsieh MH. Evaluation of cytosine DNA methylation of the Biomphalaria glabratahe at shock protein 70 locus after biological and physiological stresses [Internet]. Journal of Parasitology and Vector Biology. 2015 7:10 pp. 182–193. 10.5897/JPVB2015.0207

[23] Gutiérrez MI, Siraj AK, Khaled H, Koon N, El-Rifai W, Bhatia K. CpG island methylation in Schistosoma- and non-Schistosoma-associated bladder cancer. Mod Pathol. 2004;17: 1268–74. 10.1038/modpathol.3800177 15154012

[24] Eissa S, Swellam M, El-Khouly IM, Kassim SK, Shehata H, Mansour A, et al Aberrant methylation of RARbeta2 and APC genes in voided urine as molecular markers for early detection of bilharzial and nonbilharzial bladder cancer. Cancer Epidemiol Biomarkers Prev. 2011;20: 1657–1664. 10.1158/1055-9965.EPI-11-0237 21680534

[25] Conti SL, Honeycutt J, Odegaard JI, Gonzalgo ML, Hsieh MH. Alterations in DNA Methylation May Be the Key to Early Detection and Treatment of Schistosomal Bladder Cancer. PLoS Negl Trop Dis. 2015;9: e0003696 10.1371/journal.pntd.0003696 26042665 PMC4456143

[26] Plieskatt JL, Deenonpoe R, Mulvenna JP, Krause L, Sripa B, Bethony JM, et al Infection with the carcinogenic liver fluke Opisthorchis viverrini modifies intestinal and biliary microbiome. FASEB J. 2013;27: 4572–4584. 10.1096/fj.13-232751 23925654 PMC3804743

[27] Kay GL, Millard A, Sergeant MJ, Midzi N, Gwisai R, Mduluza T, et al Differences in the faecal microbiome in schistosoma haematobium infected children vs. uninfected children. PLoS Negl Trop Dis. 2015;9 10.1371/journal.pntd.0003861 26114287 PMC4482744

[28] Silva TM, Melo ES, Lopes ACS, Veras DL, Duarte CR, Alves LC, et al Characterization of the bacterial microbiota of biomphalaria glabrata (Say, 1818) (Mollusca: Gastropoda) from Brazil. Lett Appl Microbiol. 2013;57: 19–25. 10.1111/lam.12068 23488866

[29] Liang S, Knight M, Jolly ER. Polyethyleneimine Mediated DNA Transfection in Schistosome Parasites and Regulation of the WNT Signaling Pathway by a Dominant-Negative SmMef2. PLoS Negl Trop Dis. 2013;7 10.1371/journal.pntd.0002332 23936566 PMC3723562

