# Supplementary material for: The NIH-NIAID Schistosomiasis Resource Center at the Biomedical Research Institute: Molecular Redux
Source: PLoS Negl Trop Dis. 2016 Oct 20;10(10):e0005022. doi: 10.1371/journal.pntd.0005022 (PMC5072641; doi:10.1371/journal.pntd.0005022)
Supplement: S1 File — Investigators wishing to use SRC-generated life cycle and molecular reagents must first register on the BEI website. Included are blank registration forms for Biosafety Level 1 and 2 laboratories. (PDF) [file pntd.0005022.s001.pdf]

bei RESOURCES
SUPPORTING INFECTIOUS DISEASE RESEARCH

Level 2 Individual Registration Application Instructions

The BEI Resources Level 2 registration is for receiving Pathogen or Permit-Related Reagents which require Biosafety Level 2 rated laboratory space. The Level 2 registration does not include access to Select Agents or Biosafety Level 3 items; however, it will include access to all Level 1 and 2 reagents. The following documents must be submitted to begin the process for obtaining a Level 2 Individual Registration.

1. **BEI Resources Registration Application:** The registration application must be completed and signed by the registrant and countersigned by the Program Manager or Department Head. As the registrant, if you are the Program Manager or Department Head, please have your Supervisor act as your signatory.
   - **Special instructions for Federal Permits and Related Documents**: Please note that the address you list as your **shipping address** on the application must match any Federal Permits that you may have or may need to obtain at the time of ordering. If the address on your Federal permits does not match your registered address, then your order will be delayed until the matter is resolved.
   - **Please note**: The possession, use and transfer of biological materials are tightly controlled by federal regulations and several government agencies. A complete review of the compliance requirements is important for researchers who work with such materials.
2. **Individual Material Transfer Agreement:** The Individual Material Transfer Agreement (Individual MTA) must be completed and signed by the registrant and countersigned by a duly authorized signatory capable of legally binding your institution.
3. **Biographical Sketch of Registrant/Applicant:** The sketch, or brief curriculum vitae, must include the educational background, past and present employers and job titles, and a listing of representative publications. Include relevant institutional, departmental or laboratory web site addresses where available and applicable.
4. **A Profile of Your Institution or Company:** On your organization's letterhead, provide a brief description of your institution or company, including a web site address.
5. **Laboratory Facility Description and Biosafety Containment Level of Facility:** The Biosafety Level 2 Checklist must be completed and signed by the organization's Biosafety Officer. The form must be filled out to confirm your lab facilities, access policies, material handling procedures, and training practices fulfill the criteria established for the biosafety level guidelines of the CDC/NIH in *Biosafety in Microbiological and Biomedical Laboratories,* 5th ed. HHS Publication No. (CDC) 93-8395. U.S. Department of Health and Human Services, Centers for Disease Control. Washington DC: U.S. Government Printing Office; 2007. The entire text is available online. If more than one **shipping address** is submitted, a Biosafety Level 2 Checklist for each address is required.

BEI Resources
10801 University Blvd
Manassas, VA 20110-2209 USA
www.beiresources.org
800-359-7370
Fax: 703-365-2898
E-mail: contact@beiresources.org
Doc ID: 27128 Effective Date: 04/20/2012
Revision 3.0

6. **Customer Acceptance of Responsibility Form:** The Customer Acceptance of Responsibility Form (CAR) must be completed and signed by the registrant and countersigned by an Institutional Official.

   - **Special instructions for the BEI Resources CAR form**: Please note that the address listed on the BEI Resources CAR form needs to match your **shipping address** as listed on the application. If more than one **shipping address** is submitted, a CAR form for each address is required.

Submission Instructions:

BEI Resources can accept registration documents by email, fax or postal mail:

Email: contact@beiresources.org

Fax: (703) 365-2898

BEI Resources
Customer Services: Registration
10801 University Blvd
Manassas, VA 20110-2209
U.S.A.

Missing documents will result in a delay in the processing/approval of your application. Please allow two to three weeks after receipt of all registration materials for approval by BEI Resources and NIAID. The below documents must be submitted for Level 2 Individual Registrations.

Document	Submission Requirements
Application	One application
Material Transfer Agreement (MTA)	One MTA
Biographical Sketch of Registrant	One Biographical Sketch
Profile of Your Institution or Company	One Profile of your institution or Company
Laboratory Facility Checklist and Biosafety Containment Level (BSL) of Facility	One Level 2 Laboratory Facility Checklist and Biosafety Containment Level (BSL) of Facility (note: if multiple shipping addresses are requested, each shipping address must have a Checklist submitted).
Customer Acceptance of Responsibility (CAR)	One Customer Acceptance of Responsibility form (note: if multiple shipping addresses are requested, each shipping address must have a CAR form submitted).

If you have questions about the application process, please contact BEI Resources Customer Services either by phone, at **(800) 359-7370** or by E-mail **contact@beiresources.org**

bei RESOURCES

SUPPORTING INFECTIOUS DISEASE RESEARCH

Level 2 Registration Application

All registrations are subject to approval by BEI Resources and the National Institute of Allergy and Infectious Diseases. *Instructions: Print or type information* ***in ink*** *to be legible. Please provide all requested information. Missing information will delay the approval process or may result in denial of registration.*

**Registrant Information**		
First Name	Last Name	
Department	Building	Room Number
Street Address		City
State/Province	Zip/Postal Code	Country
Telephone	Fax	E-mail

**Alternate Contact Information**		
Last Name	First Name	E-mail

**Organization / Institution Information**			
Organization Name		Employer Identification Number (EIN)	
Street Address/P.O. Box (If different from Registrant Address)		City	
State/Province	Zip/Postal Code	Country	
Department	Web Site Address		
Check type of organization:			
☐ University/Education<br>☐ Research Foundation<br>☐ U.S. Government<br>☐ Hospital/Clinic	☐ Diagnostic Lab<br>☐ Pharmaceutical/Drug Discovery<br>☐ Biotechnology/Life Science<br>☐ Contract Laboratory	☐ Industrial<br>☐ Manufacturing<br>☐ Food Processing/Agriculture<br>☐ Environmental	☐ International Government<br>☐ Other, explain:<br>________________________<br>________________________
ATCC Account Number (if applicable)	☐ Check here if you work at a private, nonprofit 501(c)(3) organization.		

**Biosafety Officer or Environmental Officer Contact Information**		
First Name	Last Name	
Telephone	Fax	E-mail

**Shipping Address**		
Department	Building	Room Number
Street Address (PO Boxes cannot be accepted)		City
State/Province	Zip/Postal Code	Country
Telephone	Fax	E-mail

Secondary Shipping Address (Optional for Institutions with Other Designated Shipping Addresses)		
Department	Building	Room Number
Street Address (PO Boxes cannot be accepted)		City
State/Province	Zip/Postal Code	Country
Telephone	Fax	E-mail

If additional Shipping Addresses are required, please include additional pages with Shipping Address information, a Biosafety Checklist and a CAR form for each shipping address.

Scope of Use of Materials
Include a scope of use describing the work and the proposed use of the reagent(s) within that scope. Please include, if applicable, the U.S. Government grant, cooperative agreement or contract supporting that work. Please attach additional pages if needed.

Level 2 Registration Application

Please use the below checklist to ensure the additional required information is included in your registration submission.

Additional Required Information
☐ **Material Transfer Agreement (MTA)**<br>The MTA must be completed and signed by the registrant and countersigned by a duly authorized signatory capable of legally binding the institution. The MTA can be found here. New registrants at institutions with Institutional MTAs on file with BEI Resources may leverage existing agreements by submitting the Acknowledgement of the MTA form. Please contact BEI Resources at contact@beiresources.org to confirm whether an Institutional MTA is already in place between BEI Resources and your institution.
☐ **Biographical Sketch of Registrant**<br>A brief curriculum vitae or the sketch from a recent NIH grant proposal may be submitted.
☐ **Profile of Your Institution or Company**<br>On your organization's letterhead, provide a brief description of your institution or company, including a Web site address.
☐ **Laboratory Facility Checklist and Biosafety Containment Level (BSL) of Facility**<br>Please provide a signed Biosafety Level 2 checklist describing in detail how your lab facilities, access policies, material handling procedures and training practices fulfill the criteria established for biosafety level guidelines by the CDC/NIH in *Biosafety in Microbiological and Biomedical Laboratories* (BMBL), 5th edition, HHS Publication # (CDC) 93-8395. U.S. Department of Health and Human Services, Centers for Disease Control, Washington, D.C. Government Printing Office, February 2007. This checklist must be signed by the institution's biosafety officer or environmental safety officer. If more than one **shipping address** is submitted, a Biosafety Level 2 Checklist for each address is required.
☐ **Customer Acceptance of Responsibility (CAR)**<br>Registrant will also have to complete and return a signed CAR form. A copy of the CAR form can be found here. If more than one **shipping address** is submitted, a CAR form for each address is required.

We acknowledge that the information listed in this application and the required documents is current, complete and accurate to the best of our knowledge. We have read the BEI Resources Material Transfer Agreement and understand the terms and conditions of receiving materials from BEI Resources.

______________________________<br>Registrant's Name (print)	______________________________<br>Department Head/Program Director's Name (Print)
______________________________<br>Registrant's Title	______________________________<br>Department Head/Program Director's Title
______________________________<br>Registrant's Signature Date	______________________________<br>Department Head/Program Director's Signature Date

BEI Resources can accept registration documents by email, fax or postal mail. Please send this application with all required documents to:

Email: contact@beiresources.org

Fax: (703) 365-2898

BEI Resources
Customer Service: Registration
10801 University Blvd.
Manassas, VA 20110-2209

Please allow 2-3 weeks after receipt of documents for registration review and establishment of an account. Once your application is approved you will receive an email describing the services provided by BEI Resources including your username and password. Requests for materials cannot be considered until the registration process is complete. ***Thank you for your application to BEI Resources.***

If you have questions about this application or related documents, contact us at contact@beiresources.org or call 800-359-7370.

bei RESOURCES
SUPPORTING INFECTIOUS DISEASE RESEARCH

Laboratory Information: Biosafety Level 2 Checklist

All registrations are subject to approval by BEI Resources and the National Institute of Allergy and Infectious Diseases.

*Instructions: Print or type information **in ink** to be legible. Please provide all requested information. Missing information will delay the approval process or may result in denial of registration.*

**Organization / Institution Information and Address** (Please provide address where laboratory is located)		
Organization Name		
Biosafety Officer or Environmental Officer (first & last name)		
Department	Building	Room Number
BSL-2 Laboratory Address		City
State/Province	Zip/Postal Code	Country

Biosafety Level 2 is suitable for work involving agents that pose moderate hazards to personnel and the environment. It differs from BSL-1 in that 1) laboratory personnel have specific training in handling pathogenic agents and are supervised by scientists competent in handling infectious agents and associated procedures; 2) access to the laboratory is restricted when work is being conducted; and 3) all procedures in which infectious aerosols or splashes may be created are conducted in BSCs or other physical containment equipment.

This checklist is to be filled out, **initialed** (Sections A-D) and signed by the Biosafety Officer of the organization/institution.
The following standard practices, safety equipment, and facility requirements apply to BSL-2.

**A. Standard Microbiological Practices**	**YES**	**NO**	**Biosafety Officer Initials**
Are institutional policies in place and enforced for the following:<br>1. Controlled access to the laboratory;<br>2. Hand washing;<br>3. No eating, drinking, smoking, handling contact lenses, applying cosmetics, and storing food or beverages for consumption;<br>4. Mouth pipetting is prohibited;<br>5. Polices for the safe handling of sharps; and<br>6. Procedures for minimization of splashes and aerosols?			x_______
Are decontamination procedures in place and enforced for the following:<br>1. Work surfaces;<br>2. Spills involving potentially hazardous material; and<br>3. Waste generated that may contain potentially hazardous material?			x_______
Are signs posted to notify workers and others entering the laboratory of potential hazards and who is responsible for the laboratory?			x_______
Is there an effective pest management program in place?			x_______
Are laboratory personnel trained prior to assuming their duties and on an annual basis or when policies change on the hazards associated with the material they are manipulating and the precautions to prevent exposures, and exposure evaluation procedures?			x_______
**B. Special Practices**	**YES**	**NO**	**Biosafety Officer Initials**
Are all persons entering the laboratory advised of entry/exit requirements?			x_______
Does your organization have an occupational health and medical surveillance program which includes offering appropriate immunizations for the agents handled?			x_______
Is a baseline serum sample stored for laboratory employees?			x_______

Is there a biosafety manual available that is laboratory specific?			x_______
Does the laboratory supervisor ensure that all personnel working with BSL-2 agents have the appropriate knowledge and can demonstrate proficiency in standard and special laboratory practices?			x_______
Are there animals or plants in the laboratory not associated with the work being performed?			x_______

**C. Safety Equipment (Primary Barriers and Personal Protective Equipment)**
Biosafety cabinets are required for the manipulation of infectious materials. Please indicate type of BSC and certification schedule.
What personal protective equipment is required by your laboratory for the manipulation of infectious materials?

**D. Laboratory Facilities (Secondary Barriers)**	**YES**	**NO**	**Biosafety Officer Initials**
Are doors present for access control to laboratory areas?			x_______
Is a sink available for hand washing?			x_______
Is an eyewash station readily available?			x_______
When vacuum lines are used are they protected with HEPA filters?			x_______
Is the laboratory designed for easy cleaning?			x_______
Is furniture provided that is suitable for the laboratory work performed?			x_______
Are work benches provided that are impervious to water, resistant to heat, organic solvents, acids, alkalis, and other chemicals?			x_______
Are chairs and other laboratory equipment made so that they can be easily decontaminated with the appropriate disinfectant?			x_______
Does your laboratory have windows that open to the outside? If 'yes', are they fitted with screens?			x_______

Please list method of waste disposal: ____

____

Are there any additional safety features about your facilities that you would like to provide?

____

____

____

SIGNATURES ON NEXT PAGE

I acknowledge that the information listed in this facility description is current, complete and accurate to the best of my knowledge. I understand the hazards associated with the material I am requesting and I will work with it under the appropriate laboratory containment as determined by our institutional policies and procedures.

____	____
Biosafety Officer's Name (Print)	Biosafety Officer's Signature and Date

BEI Resources can accept registration documents by email, fax or postal mail. Please send documents to:

Email: contact@beiresources.org

Fax: (703) 365-2898

BEI Resources
Customer Service: Registration
10801 University Boulevard
Manassas, VA 20110-2209

If you have questions about this application or related documents, contact us at contact@beiresources.org or call **(800) 359-7370**.

bei RESOURCES
SUPPORTING INFECTIOUS DISEASE RESEARCH

INDIVIDUAL MATERIAL TRANSFER AGREEMENT

This BEI Resources Material Transfer Agreement ("BEI Resources MTA") is between the RECIPIENT and the American Type Culture Collection ("ATCC®"), a not-for-profit organization having its offices at 10801 University Blvd. Manassas, Virginia 20110 which, in its capacity as contractor to the National Institute of Allergy and Infectious Disease ("NIAID"), an Institute of the National Institutes of Health ("NIH"), an agency of the U.S. Department of Health and Human Services ("HHS"), is managing BEI Resources. BEI Resources is a U.S. Government-funded program, which is separate and distinct from other collections at the ATCC.

TERMS AND CONDITIONS

DEFINITIONS

COLLABORATOR: Any third party, other than a CONTRIBUTOR, collaborating with RECIPIENT on a project funded by the same U.S. Government grant, cooperative agreement or contract as RECIPIENT, for the purpose(s) and during the term of that funding agreement, provided that party is also registered with ATCC for access to BEI Resources and has executed a BEI Resources MTA and INTER-REGISTRANT TRANSFER AGREEMENT for that MATERIAL.

COMMERCIAL PURPOSES: For purposes of this Agreement:

(1) The term COMMERCIAL PURPOSES means the sale, license, lease, export, transfer or other distribution of MATERIAL or MODIFICATIONS to a third party for financial gain or other commercial purposes and/or the use of MATERIAL: (a) to provide a service to a third party for financial gain; (b) to produce or manufacture products for general sale or products for use in the manufacture of products ultimately intended for general sale; (c) in connection with ADME (Absorption, Distribution, Metabolism and Excretion) testing; (d) in connection with drug potency or toxicity testing which does not include either screening multiple cell lines for potential inclusion in a screening assay system or screening multiple compounds in a system for internal research purposes only; (e) in connection with proficiency testing service(s), including but not limited to, providing the service of determining laboratory performance by means of comparing and evaluating calibrations or tests on the same or similar items or materials in accordance with predetermined conditions; or (f) for research conducted under an agreement wherein a for-profit entity receives a right whether actual or contingent to the results of the research.

(2) The term COMMERCIAL PURPOSES does NOT include the use of MATERIAL or MODIFICATIONS for Industry Sponsored Academic Research or by any organization for a project funded by the U.S. Government through a grant, cooperative agreement or contract, and only for the purpose(s) of and during the term of that funding agreement. In any event, commercial sale of any product or service based on MATERIAL will require a commercial license from the CONTRIBUTOR if required by the CONTRIBUTOR at the time of deposit.

CONTRIBUTOR: Organization and/or individual providing ORIGINAL MATERIAL to ATCC for deposit into BEI Resources. CONTRIBUTOR retains ownership rights to MATERIAL where applicable as defined on the BEI Resources Deposit form.

CONTRIBUTOR LICENSE: Separate agreement between CONTRIBUTOR and RECIPIENT stating terms and conditions for use of MATERIAL and/or MODIFICATIONS, as appropriate, for COMMERCIAL PURPOSES.

DULY AUTHORIZED SIGNATORY: An individual who is authorized to execute a binding document on behalf of the RECIPIENT institution.

INTER-REGISTRANT TRANSFER AGREEMENT An agreement used by sending and receiving institutions to execute preauthorized 3rd party transfers of BEI materials and modifications. U.S. Government grant, cooperative agreement or contract number under which MATERIAL is to be used, if any, and an updated abstract of proposed use, are to be submitted to BEI Resources in connection with submitting the INTER-REGISTRANT TRANSFER AGREEMENT. Note: By providing BEI Resources with current or updated government grant or contract information you significantly facilitate requests

for materials, especially when your research requirements exceed preauthorized quantities of materials.

LETTER OF TRANSFER: An acknowledgement of the MTA, as a check box on the BEI Resources website, checked by the RECIPIENT for each requested MATERIAL.

MATERIAL: ORIGINAL MATERIAL, PROGENY, and UNMODIFIED DERIVATIVES. MATERIAL shall not include MODIFICATIONS.

MODIFICATIONS: Substances created by RECIPIENT which contain/incorporate a significant or substantial portion of MATERIAL.

ORIGINAL MATERIAL: The MATERIAL provided by CONTRIBUTOR to ATCC for deposit into BEI Resources.

PROGENY: Unmodified descendant from MATERIAL, such as by way of non-limiting example: virus from virus, cell from cell, or microorganism from microorganism.

RECIPIENT: Organization receiving ORIGINAL MATERIAL from BEI Resources through ATCC.

REGISTRANT: RECIPIENT's representative receiving and using MATERIAL.

SELECT AGENT: Specifically regulated pathogens and toxins as defined in 42 C.F.R. Part 73, 9 C.F.R. 331 and 9 C.F.R. 121, including pathogens and toxins regulated by both HHS and USDA as announced in the Federal Register, and listed on the HHS Centers for Disease Control and Prevention web-site at www.cdc.gov.

UNMODIFIED DERIVATIVE: Substance created by RECIPIENT that constitutes an unmodified functional subunit or product not changed in form or character and expressed by ORIGINAL MATERIAL. Such non-limiting examples include: subclones of unmodified cell lines, purified or fractionated subsets of ORIGINAL MATERIAL, proteins expressed by DNA/RNA supplied by CONTRIBUTOR, or monoclonal antibodies secreted by a hybridoma cell line.

RECEIPT; SCOPE OF USE AND TRANSFER

Receipt. For each requested MATERIAL, RECIPIENT must agree to the LETTER OF TRANSFER, thereby acknowledging this agreed upon MTA, during the ordering process. The receiving REGISTRANT is also required to provide an updated abstract describing the proposed use of MATERIAL if the scope of use on file has changed. If MATERIAL will be used under a U.S. Government grant, cooperative agreement or contract, the REGISTRANT must identify to BEI Resources the number and title of the funding agreement in connection with submitting the request for MATERIAL.

Select Agents. To receive SELECT AGENTS, RECIPIENT must obtain necessary permits and written proof of approval from HHS (42 C.F.R. 71, 72, 73) or USDA (7 C.F.R. 331, 9 C.F.R. 121 and 122) to possess SELECT AGENTS, copies of which must be received by ATCC before MATERIAL will be shipped. RECIPIENT acknowledges that failure to comply with any laws, regulations or other requirements relating to SELECT AGENTS may result in civil and/or criminal penalties. RECIPIENT agrees to notify ATCC regarding all MODIFICATIONS of SELECT AGENTS that retain or can attain viability or bioactivity and agrees, upon request from ATCC, to deposit those MODIFICATIONS with BEI Resources through ATCC for purposes of reference. ATCC agrees that RECIPIENT may request distribution restrictions for these MODIFICATIONS.

Scope of Use. RECIPIENT may use MATERIAL and MODIFICATIONS for research purposes and in RECIPIENT's facility only. If RECIPIENT desires to use MATERIAL or MODIFICATIONS for COMMERCIAL PURPOSES and, if required to do so by CONTRIBUTOR for such COMMERCIAL PURPOSES, RECIPIENT agrees to negotiate in good faith with CONTRIBUTOR in advance of such use to establish the terms of an appropriate commercial CONTRIBUTOR LICENSE.

Inter-Registrant Transfer. An INTER-REGISTRANT TRANSFER AGREEMENT must be used to request approval for all 3rd party transfers of BEI MATERIAL. 3rd party transfer requests of BEI MATERIAL can only be made between current BEI REGISTRANTS. MATERIAL and MODIFICATIONS may be used or transferred to the facilities of COLLABORATOR subject to any restrictions in this Agreement and the applicable INTER-REGISTRANT TRANSFER AGREEMENT.

RECIPIENT AGREES THAT MATERIAL AND MODIFICATIONS WILL NOT BE USED IN HUMANS WITHOUT PRIOR WRITTEN PROOF TO ATCC OF REGULATORY APPROVAL FOR SUCH USE. Furthermore, if RECIPIENT has received such regulatory approval, RECIPIENT agrees to conduct the clinical research in accordance with all applicable laws and regulations or otherwise in accordance with U. S. Food and Drug Administration (FDA) Good Clinical Practice (International Conference on Harmonisation (ICH) E6: "Good Clinical Practice: Consolidated Guideline"; 62 C.F.R. 25, 691 (1997)).

RECIPIENT ACKNOWLEDGES THAT MATERIALS DESIGNATED AS BIOSAFETY LEVEL 2 OR 3 CONSTITUTE KNOWN PATHOGENS OR TOXINS AND THEREFORE REQUIRE APPROPRIATE FACILITIES FOR THEIR USE. RECIPIENT ALSO ACKNOWLEDGES THAT OTHER MATERIALS NOT SO DESIGNATED AND MODIFICATIONS THEREOF MAY BE PATHOGENIC UNDER CERTAIN CONDITIONS.

OWNERSHIP OF MATERIAL AND INTELLECTUAL PROPERTY

Ownership of Material. CONTRIBUTOR retains ownership rights to MATERIAL, including MATERIAL contained or incorporated in MODIFICATIONS. CONTRIBUTOR also retains rights to any intellectual property it owns in MATERIAL. RECIPIENT retains ownership of: (a) MODIFICATIONS (except that CONTRIBUTOR retains ownership rights to MATERIAL included therein) and (b) those substances created through the use of MATERIAL or MODIFICATIONS, but which do not contain MATERIAL. If (a) or (b) results from collaborative efforts of CONTRIBUTOR and RECIPIENT, joint ownership may be negotiated. RECIPIENT understands that the U.S. Government may request RECIPIENT to deposit any or all MODIFICATIONS into BEI Resources for reference purposes.

Inventions and Patents. RECIPIENT is free to file patent application(s) claiming inventions made by RECIPIENT through the use of MATERIAL or MODIFICATIONS. RECIPIENT will retain ownership of any inventions and patents or patent applications directed thereto that it makes using MATERIAL. RECIPIENT acknowledges that use of MATERIAL or MODIFICATIONS may be subject to the intellectual property rights of third parties other than CONTRIBUTOR, and ATCC MAKES NO REPRESENTATION OR WARRANTY THAT SUCH RIGHTS DO NOT EXIST. RECIPIENT shall have sole responsibility for obtaining any appropriate intellectual property license(s) required to use MATERIAL or MODIFICATIONS.

Commercial Purposes. If RECIPIENT desires to use MATERIAL or MODIFICATIONS for COMMERCIAL PURPOSES and, if required to do so by CONTRIBUTOR for such COMMERCIAL PURPOSES, RECIPIENT agrees to negotiate in good faith with CONTRIBUTOR in advance of such use to establish the terms of an appropriate commercial CONTRIBUTOR LICENSE. RECIPIENT also agrees to provide written proof of license to ATCC in order to receive MATERIAL. RECIPIENT understands that CONTRIBUTOR shall have no obligation to grant such a CONTRIBUTOR LICENSE to RECIPIENT.

Trademarks. Nothing in this Agreement shall be construed to affect ATCC's rights, title and interests in and to trademarks registered or owned by the ATCC or the U.S. Government and any and all ATCC catalog numbers or ATCC-specific designations of biological materials sold by the ATCC.

Dispute Resolution. Any disputes arising under this Agreement shall be tried exclusively in the United States District Court for the Eastern District of Virginia or if subject matter jurisdiction does not exist in that court, then in the state courts of Virginia for Prince William County, and RECIPIENT hereby expressly consents to, submits to and waives any objection to the jurisdiction of such courts; provided however, if RECIPIENT is a US Federal or State non-profit organization; then any disputes arising under this Agreement shall be tried exclusively in a court of competent jurisdiction.

CONFIDENTIALITY; PUBLICATIONS

RECIPIENT agrees to treat in confidence, for a period of three (3) years from the date of its disclosure, any of BEI Resources' or CONTRIBUTOR's written information about MATERIAL that is stamped "CONFIDENTIAL" except for information that was previously known to RECIPIENT or that is or becomes publicly available or which is disclosed to RECIPIENT without a confidentiality obligation. Any oral disclosures from BEI RESOURCES or CONTRIBUTOR shall be identified as confidential by notice delivered to RECIPIENT within ten (10) days after the date of oral disclosure.

RECIPIENT may publish or otherwise publicly disclose the results of the work with MATERIAL, but if RECIPIENT received confidential information from BEI Resources or CONTRIBUTOR, then only after the source of the confidential information has had thirty (30) days to review the proposed disclosure to determine whether it includes any CONFIDENTIAL information, except when a shortened time period under court order of the Freedom of Information Act, 5 U.S.C.§ 552, pertains. RECIPIENT agrees to provide a copy of all publications relating to MATERIAL or MODIFICATIONS to ATCC for deposit into BEI Resources.

In all publications and patent applications that reference MATERIAL or MODIFICATIONS, RECIPIENT agrees to acknowledge BEI Resources and the BEI Resources catalog numbers if applicable, and any CONTRIBUTOR indicated through BEI Resources as the source of ORIGINAL MATERIAL. RECIPIENT agrees that ATCC may inform CONTRIBUTOR of RECIPIENT'S identity if required to do so by law, by CONTRIBUTOR, or if MATERIAL is subject to an issued patent.

WARRANTY; WARRANTY DISCLAIMER

ATCC warrants that cells and microorganisms, excluding inactivated organisms, shall be viable upon shipment from ATCC for a period of thirty (30) days ("WARRANTY PERIOD"). ATCC warrants that MATERIAL shall meet the specifications on the certificate of analysis. The exclusive remedy for breach of this warranty is, at ATCC's option, replacement of MATERIAL. The exclusive remedy applies under the condition that RECIPIENT handles and stores MATERIAL as described in any accompanying documentation. To obtain the exclusive remedy, RECIPIENT must report the lack of viability to BEI Resources' Technical Service Department within the WARRANTY PERIOD. Any expiration date specified on shipment documentation for MATERIAL states the expected remaining useful life, but does not constitute a warranty or extend any applicable warranty period. **EXCEPT AS EXPRESSLY PROVIDED ABOVE, MATERIAL AND ANY TECHNICAL INFORMATION AND ASSISTANCE PROVIDED BY ATCC ARE PROVIDED "AS IS", WITHOUT WARRANTIES OF ANY KIND, EXPRESS OR IMPLIED, INCLUDING, BUT NOT LIMITED TO ANY IMPLIED WARRANTIES OF MERCHANTABILITY, FITNESS FOR A PARTICULAR PURPOSE, TYPICALITY, SAFETY, ACCURACY AND NON-INFRINGEMENT.**

SAFETY; COMPLIANCE WITH LAWS

EXCEPT TO THE EXTENT PROHIBITED BY LAW, RECIPIENT ASSUMES ALL RISKS AND RESPONSIBILITY IN CONNECTION WITH RECIPIENT'S RECEIPT, HANDLING, STORAGE, DISPOSAL, INTERNAL TRANSFER AND USE OF MATERIAL AND MODIFICATIONS INCLUDING WITHOUT LIMITATION TAKING ALL APPROPRIATE SAFETY AND HANDLING PRECAUTIONS TO MINIMIZE HEALTH OR ENVIRONMENTAL RISK, AS WELL AS FOR ANY ADVERSE EVENTS RESULTING FROM RECIPIENT'S VIOLATION OF THE SECURITY REQUIREMENTS OR UNAUTHORIZED DISSEMINATION OF MATERIAL AND MODIFICATIONS. RECIPIENT IS SOLELY RESPONSIBLE FOR ITS COMPLIANCE WITH ALL APPLICABLE FOREIGN AND DOMESTIC, FEDERAL, STATE AND LOCAL STATUTES, ORDINANCES, REGULATIONS AND GUIDELINES.

RECIPIENT hereby certifies that RECIPIENT shall (1) ensure that only qualified personnel work with MATERIAL and MODIFICATIONS in proper facilities; (2) provide sufficient internal security to assure access to MATERIAL and MODIFICATIONS only by those individuals authorized to work with them; (3) not transfer, export, resell, or otherwise dispose of any MATERIAL or MODIFICATIONS to any third party under any circumstances without express written authorization from ATCC and the appropriate government agencies or as explicitly provided for within this Agreement; (4) not permit access to MATERIAL or MODIFICATIONS by foreign entities or individuals when to do so would be in violation of export control laws; (5) maintain adequate insurance coverage for liability to any party that might be injured by release of MATERIAL or MODIFICATIONS; (6) comply with all applicable federal, state, or local laws and regulations pertaining to MATERIAL or MODIFICATIONS or their handling, storage, use, transportation; and (7) unless requested otherwise by ATCC, destroy all MATERIAL according to accepted practices for destruction of biohazardous material upon completion of work or expiration or termination of this Agreement, whichever occurs first.

INDEMNIFICATION

RECIPIENT assumes all liability for damages that may arise from RECIPIENT's use, storage or disposal of MATERIAL and MODIFICATIONS. To the extent permitted under Federal or State law, RECIPIENT agrees that it will indemnify and hold harmless The U.S. Government, ATCC, their suppliers and CONTRIBUTORS from any claims, cost damages, or expenses.

LIMITATION OF LIABILITY

IN NO EVENT WILL ATCC, THE U.S. GOVERNMENT OR CONTRIBUTORS BE LIABLE FOR ANY SPECIAL, INCIDENTAL OR CONSEQUENTIAL DAMAGES OF ANY KIND IN CONNECTION WITH OR ARISING OUT OF THIS BEI RESOURCES MTA, MATERIAL, AND MODIFICATIONS (WHETHER IN CONTRACT, TORT, NEGLIGENCE, STRICT LIABILITY, STATUTE OR OTHERWISE) EVEN IF ATCC HAS BEEN ADVISED OF THE POSSIBILITY OF SUCH DAMAGES. RECIPIENT AGREES THAT THE LIMITATIONS OF LIABILITY SET FORTH IN THIS AGREEMENT SHALL APPLY EVEN IF A LIMITED REMEDY PROVIDED HEREUNDER FAILS OF ITS ESSENTIAL PURPOSE.

SHIPPING

MATERIAL will be packaged and shipped in accordance with applicable laws and regulations. RECIPIENT is responsible for ensuring that all permits required for RECIPIENT to receive its order are obtained and that sufficient proof of such permits is provided to ATCC. ATCC will notify RECIPIENT when orders are submitted without the necessary permits, and RECIPIENT will have a two (2) month period after such notification to supply proof of the necessary permit(s) before an order will be cancelled. A processing fee will be charged if special processing or packaging is necessary. MATERIAL is shipped Free On Board (FOB) point of shipment, via carrier of ATCC's choice.

RECIPIENT agrees that REGISTRANT shall inform ATCC in writing of the date of receipt within five (5) working days of receiving MATERIAL undamaged or within twenty-four (24) hours of receipt or expected receipt if MATERIAL is damaged or lost. If MATERIAL is damaged or lost during shipment, ATCC will replace such MATERIAL, provided RECIPIENT has reported lost or damaged shipments to the applicable carrier and notified BEI Resources' Customer Service Department within twenty-four (24) hours.

TERMINATION

This MTA will be effective in perpetuity after the date of the last signature below unless and until terminated as provided hereunder. RECIPIENT may terminate this Agreement by written notice to ATCC at least thirty (30) days in advance of the desired date of termination. RECIPIENT understands that ATCC may terminate this Agreement at any time with written notice to RECIPIENT and REGISTRANT. On termination of this Agreement, RECIPIENT agrees that any remaining MATERIAL will be destroyed (unless requested by ATCC to return remaining MATERIAL) and to provide written proof thereof to ATCC and NIAID no later than thirty (30) days from the date of termination. Upon termination of this Agreement RECIPIENT agrees to discuss with ATCC and NIAID the disposition of MODIFICATIONS.

MISCELLANEOUS

RECIPIENT may not assign or otherwise transfer this Agreement or any rights or obligations under this Agreement, whether by operation of law or otherwise. Any attempted assignment or transfer will be void and of no force or effect. This BEI Resources MTA, all documents incorporated herein by reference, and INTER-REGISTRANT TRANSFER AGREEMENT required for subsequent transfer of MATERIAL constitute the entire agreement between ATCC and RECIPIENT with respect to MATERIAL and supercede all previous agreements or representations.

RECIPIENT agrees to comply with any additional requirements of the CONTRIBUTOR as specified for a MATERIAL in the BEI Resources catalog.

The above sections on OWNERSHIP OF MATERIAL AND INTELLECTUAL PROPERTY, CONFIDENTIALITY; PUBLICATIONS, WARRANTY; WARRANTY DISCLAIMER, INDEMNIFICATION, and LIMITATION OF LIABILITY shall survive expiration or earlier termination of this Agreement**.**

SIGNATURES ON NEXT PAGE

READ AND UNDERSTOOD BY REGISTRANT

Printed Name ________________________________ Phone Number ___________________________

Position Description ______________________________ Email _________________________________

Institution ____

Address ____

Signature ____________________________________ Date ___________________________________

THE UNDERSIGNED SIGNATORY OF RECIPIENT CERTIFIES THAT HE OR SHE HAS THE AUTHORITY TO MAKE THE ABOVE CERTIFICATIONS AND REPRESENTATIONS ON BEHALF OF RECIPIENT AND FURTHER WARRANTS THAT HE OR SHE IS LEGALLY AUTHORIZED TO ENTER INTO THIS BINDING AGREEMENT ON BEHALF OF RECIPIENT.

DULY AUTHORIZED SIGNATORY FOR RECIPIENT'S INSTITUTION

Printed Name__________________________________ Phone Number_____________________________

Position Description _______________________________ Email _________________________________

Institution ____

Address ____

Signature ____________________________________ Date ___________________________________

The completed and signed Material Transfer Agreement can be sent to BEI Resources by email, fax or postal mail:

Email: contact@beiresources.org

Fax: (703) 365-2898

BEI Resources
American Type Culture Collection
10801 University Blvd.
Manassas, VA 20110-2209 USA

ATCC® is a registered trademark of the American Type Culture Collection.

**FOR ATCC USE ONLY**	
**Signature:** ______________________	**Printed Name:** ______________________
**Date:** ______________________	**Title:** ______________________

Customer Acceptance of Responsibility

On behalf of the Institution identified below, we acknowledge that the materials requested from American Type Culture Collection (ATCC) on behalf of BEI Resources represent a potential hazard to the public health and/or agricultural crops or products, including but not limited to viable microorganisms or toxins that may cause human disease. This Customer Acceptance of Responsibility (CAR) acknowledges the shipping address provided below meets all certifications on this form. Note: If multiple shipping addresses are needed, a CAR form must be submitted for each address.

PLEASE PRINT

Institution: ____

Department: ____

Shipping Address: ____

City: ____

State: ______________________ Postal code: ______________

Country: ____

Telephone: ________________ Fax: ______________________

On behalf of the Institution, we hereby certify that our Institution shall: (1) ensure that only qualified scientists work with these materials in proper facilities; (2) provide sufficient internal security to assure access to these materials only by those individuals authorized to work with them; (3) not transfer, export, resell, or otherwise dispose of any items supported by this statement to any third party under any circumstances without express written authorization from ATCC and the appropriate government agencies; (4) not permit access to the materials by foreign nationals when to do so would constitute a violation of export control laws; (5) maintain adequate insurance coverage for liability to any party that might be injured by release of these materials; (6) comply with all applicable federal, state, or local laws and regulations pertaining to these materials or their handling, storage, use, transportation; and (7) destroy all materials according to accepted practices for destruction of microbiological cultures or reagents upon completion of work.

We understand that by providing this signed form to ATCC on behalf of BEI Resources we are accepting responsibility for these agents and all risks associated with handling of them in our facility, as well as any adverse events resulting from our violation of the security requirements or unauthorized dissemination of the agents.

Print Full Name of Registrant: ____

Signature: ________________________________ Date: ______________

The undersigned officer of the Institution certifies that he or she has authority to make the above certifications and representations on behalf of the Institution and further warrants that he or she is legally authorized to enter into binding agreements on behalf of the Institution.

Print Full Name of Authorized Institutional Official: ______________________________

Signature: ________________________________ Date: ______________

Title: ____

Level 2 Institutional Registration Application Instructions

The BEI Resources Level 2 Institutional Registration is for entities who wish to register more than one researcher for access to BEI Resources Pathogen or Permit-Related Reagents. The Level 2 registration does not include access to Select Agents or Biosafety Level 3 items; however, it will include access to all Level 1 and 2 reagents. The following documents must be submitted to begin the process for obtaining a Level 2 Institutional Registration.

1. **BEI Resources Registration Application:** The registration application must be completed and signed by the registrant and countersigned by the Program Manager or Department Head. As the registrant, if you are the Program Manager or Department Head, please have your Supervisor act as your signatory. Additional researchers included in the registration should be listed on the Additional Registrant Appendix (page 4 of the application).
   - **Special instructions for Federal Permits and Related Documents:** Please note that the address you list as your **shipping address** on the application must match any Federal Permits that you may have or may need to obtain at the time of ordering. If the address on your Federal permits does not match your registered address, then your order will be delayed until the matter is resolved.
   - **Please note:** The possession, use and transfer of biological materials are tightly controlled by federal regulations and several government agencies. A complete review of the compliance requirements is important for researchers who may work with such materials.
2. **Institutional Material Transfer Agreement:** The Institutional Material Transfer Agreement (Institutional MTA) must be completed and counter signed by a duly authorized signatory capable of legally binding the institution. All registrants need to complete and sign the Acknowledgement of the Institutional Material Transfer Agreement (Page 7 of the Institutional MTA).
3. **Biographical Sketch of Each Registrant/Applicant:** The sketch, or brief curriculum vitae, must include the educational background, past and present employers and job titles, and a listing of representative publications. Include relevant institutional, departmental or laboratory web site addresses where available and applicable.
4. **Profile of the Institution or Company:** On the organization's letterhead, provide a brief description of the institution or company, including a web site address.
5. **Laboratory Facility Description and Biosafety Containment Level of Facility:** The Biosafety Level 2 Checklist must be completed and signed by the organization's Biosafety Officer. The form must be filled out to confirm your lab facilities, access policies, material handling procedures, and training practices fulfill the criteria established for the biosafety level guidelines of the CDC/NIH in *Biosafety in Microbiological and Biomedical Laboratories,* 5th ed. HHS Publication No. (CDC) 93-8395. U.S. Department of Health and Human Services, Centers for Disease Control. Washington DC: U.S. Government Printing Office; 2007. The entire text is available online. If more than one **shipping address** is submitted, a Biosafety Level 2 Checklist for each address is required.

6. **Customer Acceptance of Responsibility Form:** The Customer Acceptance of Responsibility Form (CAR) must be completed and signed by each registrant and countersigned by an Institutional Official.
   - **Special instructions for the BEI Resources CAR form**: Please note that the address listed on the BEI Resources CAR from needs to match your **shipping address** as listed on the application. If more than one **shipping address** is submitted, a CAR form for each address is required.

Submission Instructions:

BEI Resources can accept registration documents by email, fax or postal mail:

Email: contact@beiresources.org

Fax: (703) 365-2898

BEI Resources
Customer Services: Registration
10801 University Blvd
Manassas, VA 20110-2209
U.S.A.

Missing documents will result in a delay in the processing/approval of your application. Please allow two to three weeks after receipt of all registration materials for approval by BEI Resources and NIAID. The below documents must be submitted for Level 2 Institutional Registrations.

Document	Submission Requirements
Application	One application with Additional Registrant Appendix filled out with individuals who will also be fully registered.
Material Transfer Agreement (MTA)	One MTA signed by Duly Authorized Official and Acknowledgments of the MTA signed by each individual listed in the Additional Registrant Appendix in the application.
Biographical Sketch of Registrant	Additional Biographical Sketches must be submitted for each individual listed in the Additional Registrant Appendix in the application.
Profile of Your Institution or Company	One Profile of your institution or Company
Laboratory Facility Checklist and Biosafety Containment Level (BSL) of Facility	One Level 2 Laboratory Facility Checklist and Biosafety Containment Level (BSL) of Facility (note: if multiple shipping addresses are requested, each shipping address must have a Checklist submitted).
Customer Acceptance of Responsibility (CAR)	Additional Customer Acceptance of Responsibility (CAR) forms must be submitted for each individual listed in the Additional Registrant Appendix in the application. (note: if multiple shipping address are requested, each shipping address must have a CAR form submitted).

If you have questions about the application process, please contact BEI Resources Customer Services either by phone, at **(800) 359-7370** or by E-mail contact@beiresources.org

bei RESOURCES
SUPPORTING INFECTIOUS DISEASE RESEARCH

Level 2 Registration Application

All registrations are subject to approval by BEI Resources and the National Institute of Allergy and Infectious Diseases. *Instructions: Print or type information* ***in ink*** *to be legible. Please provide all requested information. Missing information will delay the approval process or may result in denial of registration.*

**Registrant Information**		
First Name	Last Name	
Department	Building	Room Number
Street Address		City
State/Province	Zip/Postal Code	Country
Telephone	Fax	E-mail

**Alternate Contact Information**		
Last Name	First Name	E-mail

**Organization / Institution Information**			
Organization Name		Employer Identification Number (EIN)	
Street Address/P.O. Box (If different from Registrant Address)		City	
State/Province	Zip/Postal Code	Country	
Department	Web Site Address		
Check type of organization:			
☐ University/Education<br>☐ Research Foundation<br>☐ U.S. Government<br>☐ Hospital/Clinic	☐ Diagnostic Lab<br>☐ Pharmaceutical/Drug Discovery<br>☐ Biotechnology/Life Science<br>☐ Contract Laboratory	☐ Industrial<br>☐ Manufacturing<br>☐ Food Processing/Agriculture<br>☐ Environmental	☐ International Government<br>☐ Other, explain:<br>________________________<br>________________________
ATCC Account Number (if applicable)	☐ Check here if you work at a private, nonprofit 501(c)(3) organization.		

**Biosafety Officer or Environmental Officer Contact Information**		
First Name	Last Name	
Telephone	Fax	E-mail

**Shipping Address**		
Department	Building	Room Number
Street Address (PO Boxes cannot be accepted)		City
State/Province	Zip/Postal Code	Country
Telephone	Fax	E-mail

Secondary Shipping Address (Optional for Institutions with Other Designated Shipping Addresses)		
Department	Building	Room Number
Street Address (PO Boxes cannot be accepted)		City
State/Province	Zip/Postal Code	Country
Telephone	Fax	E-mail

If additional Shipping Addresses are required, please include additional pages with Shipping Address information, a Biosafety Checklist and a CAR form for each shipping address.

Scope of Use of Materials
Include a scope of use describing the work and the proposed use of the reagent(s) within that scope. Please include, if applicable, the U.S. Government grant, cooperative agreement or contract supporting that work. Please attach additional pages if needed.

BEI RESOURCES
SUPPORTING INFECTIOUS DISEASE RESEARCH

Level 2 Registration Application

Please use the below checklist to ensure the additional required information is included in your registration submission.

Additional Required Information
☐ **Material Transfer Agreement (MTA)**<br>The MTA must be completed and signed by the registrant and countersigned by a duly authorized signatory capable of legally binding the institution. The MTA can be found here. New registrants at institutions with Institutional MTAs on file with BEI Resources may leverage existing agreements by submitting the Acknowledgement of the MTA form. Please contact BEI Resources at contact@beiresources.org to confirm whether an Institutional MTA is already in place between BEI Resources and your institution.
☐ **Biographical Sketch of Registrant**<br>A brief curriculum vitae or the sketch from a recent NIH grant proposal may be submitted.
☐ **Profile of Your Institution or Company**<br>On your organization's letterhead, provide a brief description of your institution or company, including a Web site address.
☐ **Laboratory Facility Checklist and Biosafety Containment Level (BSL) of Facility**<br>Please provide a signed Biosafety Level 2 checklist describing in detail how your lab facilities, access policies, material handling procedures and training practices fulfill the criteria established for biosafety level guidelines by the CDC/NIH in *Biosafety in Microbiological and Biomedical Laboratories* (BMBL), 5th edition, HHS Publication # (CDC) 93-8395. U.S. Department of Health and Human Services, Centers for Disease Control, Washington, D.C. Government Printing Office, February 2007. This checklist must be signed by the institution's biosafety officer or environmental safety officer. If more than one **shipping address** is submitted, a Biosafety Level 2 Checklist for each address is required.
☐ **Customer Acceptance of Responsibility (CAR)**<br>Registrant will also have to complete and return a signed CAR form. A copy of the CAR form can be found here. If more than one **shipping address** is submitted, a CAR form for each address is required.

We acknowledge that the information listed in this application and the required documents is current, complete and accurate to the best of our knowledge. We have read the BEI Resources Material Transfer Agreement and understand the terms and conditions of receiving materials from BEI Resources.

______________________________	______________________________
Registrant's Name (print)	Department Head/Program Director's Name (Print)
______________________________	______________________________
Registrant's Title	Department Head/Program Director's Title
______________________________	______________________________
Registrant's Signature Date	Department Head/Program Director's Signature Date

BEI Resources can accept registration documents by email, fax or postal mail. Please send this application with all required documents to:

Email: contact@beiresources.org

Fax: (703) 365-2898

BEI Resources
Customer Service: Registration
10801 University Blvd.
Manassas, VA 20110-2209

Please allow 2-3 weeks after receipt of documents for registration review and establishment of an account. Once your application is approved you will receive an email describing the services provided by BEI Resources including your username and password. Requests for materials cannot be considered until the registration process is complete. ***Thank you for your application to BEI Resources.***

If you have questions about this application or related documents, contact us at contact@beiresources.org or call 800-359-7370.

Additional Registrant Appendix

BEI Resources offers institutions with multiple researchers the ability to register under one registration package. Additional registrants must identify themselves below and must be working in the same laboratory or for the same entity as the primary registrant. The institution must submit an Institutional MTA to bind all researchers.

All registrations are subject to approval by BEI Resources and the National Institute of Allergy and Infectious Diseases.
*Instructions: Print or type information **in ink** to be legible. Please provide all requested information. Missing information will delay the approval process or may result in denial of registration.*

**Additional Registrants Covered in Application**		
First Name	Last Name	
Department	Building	Room Number
Street Address		City
State/Province	Zip/Postal Code	Country
Telephone	Fax	E-mail
Title	Signature and Date	
Alternate Contact Name:		Email

**Additional Registrants Covered in Application**		
First Name	Last Name	
Department	Building	Room Number
Street Address		City
State/Province	Zip/Postal Code	Country
Telephone	Fax	E-mail
Title	Signature and Date	
Alternate Contact Name:		Email

**Additional Registrants Covered in Application**		
First Name	Last Name	
Department	Building	Room Number
Street Address		City
State/Province	Zip/Postal Code	Country
Telephone	Fax	E-mail
Title	Signature and Date	
Alternate Contact Name:		Email

Please allow 2-3 weeks after receipt of documents for registration review and establishment of an account. Once your application is approved you will receive an email describing the services provided by BEI Resources including your username and password. Requests for materials cannot be considered until the registration process is complete. ***Thank you for your application to BEI Resources.***

If you have questions about this application or related documents, contact us at contact@beiresources.org or call 800-359-7370.

Laboratory Information: Biosafety Level 2 Checklist

bei RESOURCES
SUPPORTING INFECTIOUS DISEASE RESEARCH

All registrations are subject to approval by BEI Resources and the National Institute of Allergy and Infectious Diseases.

*Instructions: Print or type information **in ink** to be legible. Please provide all requested information. Missing information will delay the approval process or may result in denial of registration.*

**Organization / Institution Information and Address** (Please provide address where laboratory is located)		
Organization Name		
Biosafety Officer or Environmental Officer (first & last name)		
Department	Building	Room Number
BSL-2 Laboratory Address		City
State/Province	Zip/Postal Code	Country

Biosafety Level 2 is suitable for work involving agents that pose moderate hazards to personnel and the environment. It differs from BSL-1 in that 1) laboratory personnel have specific training in handling pathogenic agents and are supervised by scientists competent in handling infectious agents and associated procedures; 2) access to the laboratory is restricted when work is being conducted; and 3) all procedures in which infectious aerosols or splashes may be created are conducted in BSCs or other physical containment equipment.

This checklist is to be filled out, **initialed** (Sections A-D) and signed by the Biosafety Officer of the organization/institution. The following standard practices, safety equipment, and facility requirements apply to BSL-2.

**A. Standard Microbiological Practices**	**YES**	**NO**	**Biosafety Officer Initials**
Are institutional policies in place and enforced for the following:<br>1. Controlled access to the laboratory;<br>2. Hand washing;<br>3. No eating, drinking, smoking, handling contact lenses, applying cosmetics, and storing food or beverages for consumption;<br>4. Mouth pipetting is prohibited;<br>5. Polices for the safe handling of sharps; and<br>6. Procedures for minimization of splashes and aerosols?			x_______
Are decontamination procedures in place and enforced for the following:<br>1. Work surfaces;<br>2. Spills involving potentially hazardous material; and<br>3. Waste generated that may contain potentially hazardous material?			x_______
Are signs posted to notify workers and others entering the laboratory of potential hazards and who is responsible for the laboratory?			x_______
Is there an effective pest management program in place?			x_______
Are laboratory personnel trained prior to assuming their duties and on an annual basis or when policies change on the hazards associated with the material they are manipulating and the precautions to prevent exposures, and exposure evaluation procedures?			x_______
**B. Special Practices**	**YES**	**NO**	**Biosafety Officer Initials**
Are all persons entering the laboratory advised of entry/exit requirements?			x_______
Does your organization have an occupational health and medical surveillance program which includes offering appropriate immunizations for the agents handled?			x_______
Is a baseline serum sample stored for laboratory employees?			x_______

Is there a biosafety manual available that is laboratory specific?			x_______
Does the laboratory supervisor ensure that all personnel working with BSL-2 agents have the appropriate knowledge and can demonstrate proficiency in standard and special laboratory practices?			x_______
Are there animals or plants in the laboratory not associated with the work being performed?			x_______

**C. Safety Equipment (Primary Barriers and Personal Protective Equipment)**
Biosafety cabinets are required for the manipulation of infectious materials. Please indicate type of BSC and certification schedule.
What personal protective equipment is required by your laboratory for the manipulation of infectious materials?

**D. Laboratory Facilities (Secondary Barriers)**	**YES**	**NO**	**Biosafety Officer Initials**
Are doors present for access control to laboratory areas?			x_______
Is a sink available for hand washing?			x_______
Is an eyewash station readily available?			x_______
When vacuum lines are used are they protected with HEPA filters?			x_______
Is the laboratory designed for easy cleaning?			x_______
Is furniture provided that is suitable for the laboratory work performed?			x_______
Are work benches provided that are impervious to water, resistant to heat, organic solvents, acids, alkalis, and other chemicals?			x_______
Are chairs and other laboratory equipment made so that they can be easily decontaminated with the appropriate disinfectant?			x_______
Does your laboratory have windows that open to the outside? If ‘yes’, are they fitted with screens?			x_______

Please list method of waste disposal: ____

____

Are there any additional safety features about your facilities that you would like to provide?

____

____

____

SIGNATURES ON NEXT PAGE

I acknowledge that the information listed in this facility description is current, complete and accurate to the best of my knowledge. I understand the hazards associated with the material I am requesting and I will work with it under the appropriate laboratory containment as determined by our institutional policies and procedures.

____ Biosafety Officer's Name (Print)

____ Biosafety Officer's Signature and Date

BEI Resources can accept registration documents by email, fax or postal mail. Please send documents to:

Email: contact@beiresources.org

Fax: **(703) 365-2898**

BEI Resources
Customer Service: Registration
10801 University Boulevard
Manassas, VA 20110-2209

If you have questions about this application or related documents, contact us at contact@beiresources.org or call **(800) 359-7370**.

INSTITUTIONAL
MATERIAL TRANSFER AGREEMENT

This BEI Resources Material Transfer Agreement ("BEI Resources MTA") is between the RECIPIENT and the American Type Culture Collection ("ATCC®"), a not-for-profit organization having its offices at 10801 University Blvd. Manassas, Virginia 20110 which, in its capacity as contractor to the National Institute of Allergy and Infectious Disease ("NIAID"), an Institute of the National Institutes of Health ("NIH"), an agency of the U.S. Department of Health and Human Services ("HHS"), is managing BEI Resources. BEI Resources is a U.S. Government-funded program, which is separate and distinct from other collections at the ATCC. This is a master BEI Resources MTA which will apply to RECIPIENT'S REGISTRANTs only who are registered with BEI Resources and have completed the corresponding Acknowledgement of BEI MTA page.

TERMS AND CONDITIONS

DEFINITIONS

COLLABORATOR: Any third party, other than a CONTRIBUTOR, collaborating with RECIPIENT on a project funded by the same U.S. Government grant, cooperative agreement or contract as RECIPIENT, for the purpose(s) and during the term of that funding agreement, provided that party is also registered with ATCC for access to BEI Resources and has executed a BEI Resources MTA and INTER-REGISTRANT TRANSFER AGREEMENT for that MATERIAL.

COMMERCIAL PURPOSES: For purposes of this Agreement:

(1) The term COMMERCIAL PURPOSES means the sale, license, lease, export, transfer or other distribution of MATERIAL or MODIFICATIONS to a third party for financial gain or other commercial purposes and/or the use of MATERIAL: (a) to provide a service to a third party for financial gain; (b) to produce or manufacture products for general sale or products for use in the manufacture of products ultimately intended for general sale; (c) in connection with ADME (Absorption, Distribution, Metabolism and Excretion) testing; (d) in connection with drug potency or toxicity testing which does not include either screening multiple cell lines for potential inclusion in a screening assay system or screening multiple compounds in a system for internal research purposes only; (e) in connection with proficiency testing service(s), including but not limited to, providing the service of determining laboratory performance by means of comparing and evaluating calibrations or tests on the same or similar items or materials in accordance with predetermined conditions; or (f) for research conducted under an agreement wherein a for-profit entity receives a right whether actual or contingent to the results of the research.

(2) The term COMMERCIAL PURPOSES does NOT include the use of MATERIAL or MODIFICATIONS for Industry Sponsored Academic Research or by any organization for a project funded by the U.S. Government through a grant, cooperative agreement or contract, and only for the purpose(s) of and during the term of that funding agreement. In any event, commercial sale of any product or service based on MATERIAL will require a commercial license from the CONTRIBUTOR if required by the CONTRIBUTOR at the time of deposit.

CONTRIBUTOR: Organization and/or individual providing ORIGINAL MATERIAL to ATCC for deposit into BEI Resources. CONTRIBUTOR retains ownership rights to MATERIAL where applicable as defined on the BEI Resources Deposit form.

CONTRIBUTOR LICENSE: Separate agreement between CONTRIBUTOR and RECIPIENT stating terms and conditions for use of MATERIAL and/or MODIFICATIONS, as appropriate, for COMMERCIAL PURPOSES.

DULY AUTHORIZED SIGNATORY: An individual who is authorized to execute a binding document on behalf of the RECIPIENT institution.

INTER-REGISTRANT TRANSFER AGREEMENT An agreement used by sending and receiving institutions to execute preauthorized 3rd party transfers of BEI materials and modifications. U.S. Government grant, cooperative agreement or contract number under which MATERIAL is to be used, if any, and an updated abstract of proposed use, are to be submitted to BEI Resources in connection with submitting the INTER-REGISTRANT TRANSFER AGREEMENT. Note: By providing BEI Resources

with current or updated government grant or contract information you significantly facilitate requests for materials, especially when your research requirements exceed preauthorized quantities of materials.

LETTER OF TRANSFER: An acknowledgement of the MTA, as a check box on the BEI Resources website, checked by the RECIPIENT for each requested MATERIAL.

MATERIAL: ORIGINAL MATERIAL, PROGENY, and UNMODIFIED DERIVATIVES. MATERIAL shall not include MODIFICATIONS.

MODIFICATIONS: Substances created by RECIPIENT which contain/incorporate a significant or substantial portion of MATERIAL.

ORIGINAL MATERIAL: The MATERIAL provided by CONTRIBUTOR to ATCC for deposit into BEI Resources.

PROGENY: Unmodified descendant from MATERIAL, such as by way of non-limiting example: virus from virus, cell from cell, or microorganism from microorganism.

RECIPIENT: Organization receiving ORIGINAL MATERIAL from BEI Resources through ATCC.

REGISTRANT: RECIPIENT's representative receiving and using MATERIAL.

SELECT AGENT: Specifically regulated pathogens and toxins as defined in 42 C.F.R. Part 73, 9 C.F.R. 331 and 9 C.F.R. 121, including pathogens and toxins regulated by both HHS and USDA as announced in the Federal Register, and listed on the HHS Centers for Disease Control and Prevention web-site at www.cdc.gov.

UNMODIFIED DERIVATIVE: Substance created by RECIPIENT that constitutes an unmodified functional subunit or product not changed in form or character and expressed by ORIGINAL MATERIAL. Such non-limiting examples include: subclones of unmodified cell lines, purified or fractionated subsets of ORIGINAL MATERIAL, proteins expressed by DNA/RNA supplied by CONTRIBUTOR, or monoclonal antibodies secreted by a hybridoma cell line.

RECEIPT; SCOPE OF USE AND TRANSFER

Receipt. For each requested MATERIAL, RECIPIENT must agree to the LETTER OF TRANSFER, thereby acknowledging this agreed upon MTA, during the ordering process. The receiving REGISTRANT is also required to provide an updated abstract describing the proposed use of MATERIAL if the scope of use on file has changed. If MATERIAL will be used under a U.S. Government grant, cooperative agreement or contract, the REGISTRANT must identify to BEI Resources the number and title of the funding agreement in connection with submitting the request for MATERIAL.

Select Agents. To receive SELECT AGENTS, RECIPIENT must obtain necessary permits and written proof of approval from HHS (42 C.F.R. 71, 72, 73) or USDA (7 C.F.R. 331, 9 C.F.R. 121 and 122) to possess SELECT AGENTS, copies of which must be received by ATCC before MATERIAL will be shipped. RECIPIENT acknowledges that failure to comply with any laws, regulations or other requirements relating to SELECT AGENTS may result in civil and/or criminal penalties. RECIPIENT agrees to notify ATCC regarding all MODIFICATIONS of SELECT AGENTS that retain or can attain viability or bioactivity and agrees, upon request from ATCC, to deposit those MODIFICATIONS with BEI Resources through ATCC for purposes of reference. ATCC agrees that RECIPIENT may request distribution restrictions for these MODIFICATIONS.

Scope of Use. RECIPIENT may use MATERIAL and MODIFICATIONS for research purposes and in RECIPIENT's facility only. If RECIPIENT desires to use MATERIAL or MODIFICATIONS for COMMERCIAL PURPOSES and, if required to do so by CONTRIBUTOR for such COMMERCIAL PURPOSES, RECIPIENT agrees to negotiate in good faith with CONTRIBUTOR in advance of such use to establish the terms of an appropriate commercial CONTRIBUTOR LICENSE.

Inter-Registrant Transfer. An INTER-REGISTRANT TRANSFER AGREEMENT must be used to request approval for all 3rd party transfers of BEI MATERIAL. 3rd party transfer requests of BEI MATERIAL can only be made between current BEI REGISTRANTS. MATERIAL and MODIFICATIONS may be used or transferred to the facilities of COLLABORATOR subject to any restrictions in this Agreement and the applicable INTER-REGISTRANT TRANSFER AGREEMENT.

RECIPIENT AGREES THAT MATERIAL AND MODIFICATIONS WILL NOT BE USED IN HUMANS WITHOUT PRIOR WRITTEN PROOF TO ATCC OF REGULATORY APPROVAL FOR SUCH USE. Furthermore, if RECIPIENT has received such regulatory approval, RECIPIENT agrees to conduct the clinical research in accordance with all applicable laws and regulations or otherwise in accordance with U. S. Food and Drug Administration (FDA) Good Clinical Practice (International Conference on Harmonisation (ICH) E6: "Good Clinical Practice: Consolidated Guideline"; 62 C.F.R. 25, 691 (1997)).

RECIPIENT ACKNOWLEDGES THAT MATERIALS DESIGNATED AS BIOSAFETY LEVEL 2 OR 3 CONSTITUTE KNOWN PATHOGENS OR TOXINS AND THEREFORE REQUIRE APPROPRIATE FACILITIES FOR THEIR USE. RECIPIENT ALSO ACKNOWLEDGES THAT OTHER MATERIALS NOT SO DESIGNATED AND MODIFICATIONS THEREOF MAY BE PATHOGENIC UNDER CERTAIN CONDITIONS.

OWNERSHIP OF MATERIAL AND INTELLECTUAL PROPERTY

Ownership of Material. CONTRIBUTOR retains ownership rights to MATERIAL, including MATERIAL contained or incorporated in MODIFICATIONS. CONTRIBUTOR also retains rights to any intellectual property it owns in MATERIAL. RECIPIENT retains ownership of: (a) MODIFICATIONS (except that CONTRIBUTOR retains ownership rights to MATERIAL included therein) and (b) those substances created through the use of MATERIAL or MODIFICATIONS, but which do not contain MATERIAL. If (a) or (b) results from collaborative efforts of CONTRIBUTOR and RECIPIENT, joint ownership may be negotiated. RECIPIENT understands that the U.S. Government may request RECIPIENT to deposit any or all MODIFICATIONS into BEI Resources for reference purposes.

Inventions and Patents. RECIPIENT is free to file patent application(s) claiming inventions made by RECIPIENT through the use of MATERIAL or MODIFICATIONS. RECIPIENT will retain ownership of any inventions and patents or patent applications directed thereto that it makes using MATERIAL. RECIPIENT acknowledges that use of MATERIAL or MODIFICATIONS may be subject to the intellectual property rights of third parties other than CONTRIBUTOR, and ATCC MAKES NO REPRESENTATION OR WARRANTY THAT SUCH RIGHTS DO NOT EXIST. RECIPIENT shall have sole responsibility for obtaining any appropriate intellectual property license(s) required to use MATERIAL or MODIFICATIONS.

Commercial Purposes. If RECIPIENT desires to use MATERIAL or MODIFICATIONS for COMMERCIAL PURPOSES and, if required to do so by CONTRIBUTOR for such COMMERCIAL PURPOSES, RECIPIENT agrees to negotiate in good faith with CONTRIBUTOR in advance of such use to establish the terms of an appropriate commercial CONTRIBUTOR LICENSE. RECIPIENT also agrees to provide written proof of license to ATCC in order to receive MATERIAL. RECIPIENT understands that CONTRIBUTOR shall have no obligation to grant such a CONTRIBUTOR LICENSE to RECIPIENT.

Trademarks. Nothing in this Agreement shall be construed to affect ATCC's rights, title and interests in and to trademarks registered or owned by the ATCC or the U.S. Government and any and all ATCC catalog numbers or ATCC-specific designations of biological materials sold by the ATCC.

Dispute Resolution. Any disputes arising under this Agreement shall be tried exclusively in the United States District Court for the Eastern District of Virginia or if subject matter jurisdiction does not exist in that court, then in the state courts of Virginia for Prince William County, and RECIPIENT hereby expressly consents to, submits to and waives any objection to the jurisdiction of such courts; provided however, if RECIPIENT is a US Federal or State non-profit organization; then any disputes arising under this Agreement shall be tried exclusively in a court of competent jurisdiction.

CONFIDENTIALITY; PUBLICATIONS

RECIPIENT agrees to treat in confidence, for a period of three (3) years from the date of its disclosure, any of BEI Resources' or CONTRIBUTOR's written information about MATERIAL that is stamped "CONFIDENTIAL" except for information that was previously known to RECIPIENT or that is or becomes publicly available or which is disclosed to RECIPIENT without a confidentiality obligation. Any oral disclosures from BEI RESOURCES or CONTRIBUTOR shall be identified as confidential by notice delivered to RECIPIENT within ten (10) days after the date of oral disclosure.

RECIPIENT may publish or otherwise publicly disclose the results of the work with MATERIAL, but if RECIPIENT received confidential information from BEI Resources or CONTRIBUTOR, then only after the source of the confidential information has had thirty (30) days to review the proposed disclosure to determine whether it includes any CONFIDENTIAL information, except when a shortened time period under court order of the Freedom of Information Act, 5 U.S.C.§ 552, pertains. RECIPIENT agrees to provide a copy of all publications relating to MATERIAL or MODIFICATIONS to ATCC for deposit into BEI Resources.

In all publications and patent applications that reference MATERIAL or MODIFICATIONS, RECIPIENT agrees to acknowledge BEI Resources and the BEI Resources catalog numbers if applicable, and any CONTRIBUTOR indicated through BEI Resources as the source of ORIGINAL MATERIAL. RECIPIENT agrees that ATCC may inform CONTRIBUTOR of RECIPIENT'S identity if required to do so by law, by CONTRIBUTOR, or if MATERIAL is subject to an issued patent.

WARRANTY; WARRANTY DISCLAIMER

ATCC warrants that cells and microorganisms, excluding inactivated organisms, shall be viable upon shipment from ATCC for a period of thirty (30) days ("WARRANTY PERIOD"). ATCC warrants that MATERIAL shall meet the specifications on the certificate of analysis. The exclusive remedy for breach of this warranty is, at ATCC's option, replacement of MATERIAL. The exclusive remedy applies under the condition that RECIPIENT handles and stores MATERIAL as described in any accompanying documentation. To obtain the exclusive remedy, RECIPIENT must report the lack of viability to BEI Resources' Technical Service Department within the WARRANTY PERIOD. Any expiration date specified on shipment documentation for MATERIAL states the expected remaining useful life, but does not constitute a warranty or extend any applicable warranty period. **EXCEPT AS EXPRESSLY PROVIDED ABOVE, MATERIAL AND ANY TECHNICAL INFORMATION AND ASSISTANCE PROVIDED BY ATCC ARE PROVIDED "AS IS", WITHOUT WARRANTIES OF ANY KIND, EXPRESS OR IMPLIED, INCLUDING, BUT NOT LIMITED TO ANY IMPLIED WARRANTIES OF MERCHANTABILITY, FITNESS FOR A PARTICULAR PURPOSE, TYPICALITY, SAFETY, ACCURACY AND NON-INFRINGEMENT.**

SAFETY; COMPLIANCE WITH LAWS

EXCEPT TO THE EXTENT PROHIBITED BY LAW, RECIPIENT ASSUMES ALL RISKS AND RESPONSIBILITY IN CONNECTION WITH RECIPIENT'S RECEIPT, HANDLING, STORAGE, DISPOSAL, INTERNAL TRANSFER AND USE OF MATERIAL AND MODIFICATIONS INCLUDING WITHOUT LIMITATION TAKING ALL APPROPRIATE SAFETY AND HANDLING PRECAUTIONS TO MINIMIZE HEALTH OR ENVIRONMENTAL RISK, AS WELL AS FOR ANY ADVERSE EVENTS RESULTING FROM RECIPIENT'S VIOLATION OF THE SECURITY REQUIREMENTS OR UNAUTHORIZED DISSEMINATION OF MATERIAL AND MODIFICATIONS. RECIPIENT IS SOLELY RESPONSIBLE FOR ITS COMPLIANCE WITH ALL APPLICABLE FOREIGN AND DOMESTIC, FEDERAL, STATE AND LOCAL STATUTES, ORDINANCES, REGULATIONS AND GUIDELINES.

RECIPIENT hereby certifies that RECIPIENT shall (1) ensure that only qualified personnel work with MATERIAL and MODIFICATIONS in proper facilities; (2) provide sufficient internal security to assure access to MATERIAL and MODIFICATIONS only by those individuals authorized to work with them; (3) not transfer, export, resell, or otherwise dispose of any MATERIAL or MODIFICATIONS to any third party under any circumstances without express written authorization from ATCC and the appropriate government agencies or as explicitly provided for within this Agreement; (4) not permit access to MATERIAL or MODIFICATIONS by foreign entities or individuals when to do so would be in violation of export control laws; (5) maintain adequate insurance coverage for liability to any party that might be injured by release of MATERIAL or MODIFICATIONS; (6) comply with all applicable federal, state, or local laws and regulations pertaining to MATERIAL or MODIFICATIONS or their handling, storage, use, transportation; and (7) unless requested otherwise by ATCC, destroy all MATERIAL according to accepted practices for destruction of biohazardous material upon completion of work or expiration or termination of this Agreement, whichever occurs first.

INDEMNIFICATION

RECIPIENT assumes all liability for damages that may arise from RECIPIENT's use, storage or disposal of MATERIAL and MODIFICATIONS. To the extent permitted under Federal or State law,

RECIPIENT agrees that it will indemnify and hold harmless The U.S. Government, ATCC, their suppliers and CONTRIBUTORS from any claims, cost damages, or expenses.

LIMITATION OF LIABILITY

IN NO EVENT WILL ATCC, THE U.S. GOVERNMENT OR CONTRIBUTORS BE LIABLE FOR ANY SPECIAL, INCIDENTAL OR CONSEQUENTIAL DAMAGES OF ANY KIND IN CONNECTION WITH OR ARISING OUT OF THIS BEI RESOURCES MTA, MATERIAL, AND MODIFICATIONS (WHETHER IN CONTRACT, TORT, NEGLIGENCE, STRICT LIABILITY, STATUTE OR OTHERWISE) EVEN IF ATCC HAS BEEN ADVISED OF THE POSSIBILITY OF SUCH DAMAGES. RECIPIENT AGREES THAT THE LIMITATIONS OF LIABILITY SET FORTH IN THIS AGREEMENT SHALL APPLY EVEN IF A LIMITED REMEDY PROVIDED HEREUNDER FAILS OF ITS ESSENTIAL PURPOSE.

SHIPPING

MATERIAL will be packaged and shipped in accordance with applicable laws and regulations. RECIPIENT is responsible for ensuring that all permits required for RECIPIENT to receive its order are obtained and that sufficient proof of such permits is provided to ATCC. ATCC will notify RECIPIENT when orders are submitted without the necessary permits, and RECIPIENT will have a two (2) month period after such notification to supply proof of the necessary permit(s) before an order will be cancelled. A processing fee will be charged if special processing or packaging is necessary. MATERIAL is shipped Free On Board (FOB) point of shipment, via carrier of ATCC's choice.

RECIPIENT agrees that REGISTRANT shall inform ATCC in writing of the date of receipt within five (5) working days of receiving MATERIAL undamaged or within twenty-four (24) hours of receipt or expected receipt if MATERIAL is damaged or lost. If MATERIAL is damaged or lost during shipment, ATCC will replace such MATERIAL, provided RECIPIENT has reported lost or damaged shipments to the applicable carrier and notified BEI Resources' Customer Service Department within twenty-four (24) hours.

TERMINATION

This MTA will be effective in perpetuity after the date of the last signature below unless and until terminated as provided hereunder. RECIPIENT may terminate this Agreement by written notice to ATCC at least thirty (30) days in advance of the desired date of termination. RECIPIENT understands that ATCC may terminate this Agreement at any time with written notice to RECIPIENT and REGISTRANT. On termination of this Agreement, RECIPIENT agrees that any remaining MATERIAL will be destroyed (unless requested by ATCC to return remaining MATERIAL) and to provide written proof thereof to ATCC and NIAID no later than thirty (30) days from the date of termination. Upon termination of this Agreement RECIPIENT agrees to discuss with ATCC and NIAID the disposition of MODIFICATIONS.

MISCELLANEOUS

RECIPIENT may not assign or otherwise transfer this Agreement or any rights or obligations under this Agreement, whether by operation of law or otherwise. Any attempted assignment or transfer will be void and of no force or effect. This BEI Resources MTA, all documents incorporated herein by reference, and INTER-REGISTRANT TRANSFER AGREEMENT required for subsequent transfer of MATERIAL constitute the entire agreement between ATCC and RECIPIENT with respect to MATERIAL and supercede all previous agreements or representations.

RECIPIENT agrees to comply with any additional requirements of the CONTRIBUTOR as specified for a MATERIAL in the BEI Resources catalog.

The above sections on OWNERSHIP OF MATERIAL AND INTELLECTUAL PROPERTY, CONFIDENTIALITY; PUBLICATIONS, WARRANTY; WARRANTY DISCLAIMER, INDEMNIFICATION, and LIMITATION OF LIABILITY shall survive expiration or earlier termination of this Agreement**.**

SIGNATURES ON NEXT PAGE

THE UNDERSIGNED SIGNATORY OF RECIPIENT CERTIFIES THAT HE OR SHE HAS THE AUTHORITY TO MAKE THE ABOVE CERTIFICATIONS AND REPRESENTATIONS ON BEHALF OF RECIPIENT AND FURTHER WARRANTS THAT HE OR SHE IS LEGALLY AUTHORIZED TO ENTER INTO THIS BINDING AGREEMENT ON BEHALF OF RECIPIENT.

REGISTRANT - Each Registrant will sign a corresponding Acknowledgement of BEI MTA page.

DULY AUTHORIZED SIGNATORY FOR RECIPIENT'S INSTITUTION

Printed Name__________________________________ Phone Number ______________________________

Position Description ________________________________ Email _______________________________________

Institution ____

Address ____

Signature_______________________________________ Date ____

FOR ATCC USE ONLY

Printed Name__________________________________

Title____

Signature_____________________________________

Date____

The completed and signed Material Transfer Agreement can be sent to BEI Resources by email, fax or postal mail:

Email: contact@beiresources.org

Fax: (703) 365-2898

BEI Resources
American Type Culture Collection
10801 University Blvd.
Manassas, VA 20110-2209 USA

ATCC® is a registered trademark of the American Type Culture Collection.

ACKNOWLEDGEMENT OF MATERIAL TRANSFER AGREEMENT

This Acknowledgement of the Material Transfer Agreement **is only** for use by institutions that have Institutional Material Transfer Agreements on file with BEI Resources. If you do not have an Institutional Material Transfer Agreement on file, you can access the document at http://www.beiresources.org/Register/RegistrationForms/tabid/261/Default.aspx.

THE REGISTRANT AND THE UNDERSIGNED SIGNATORY OF RECIPIENT CERTIFIES THAT HE OR SHE HAS READ AND AGREED TO THE TERMS AND CONDITIONS OF THE EXISTING BEI RESOURCES MATERIAL TRANSFER AGREEMENT IN PLACE WITH THE RECIPIENT INSITUTION INDICATED BELOW.

READ AND UNDERSTOOD BY REGISTRANT

Printed Name ________________________________

Position Description ________________________________

Institution ________________________________

Institution Street Address ________________________________

City/State/Postal Code ________________________________

Country ________________________________

Date __________________________

Signature __________________________

The completed and signed Material Transfer Agreement can be sent to BEI Resources by email, fax or postal mail:

Email: contact@beiresources.org

Fax: (703) 365-2898

BEI Resources
American Type Culture Collection
10801 University Blvd.
Manassas, VA 20110-2209 USA

ATCC® is a registered trademark of the American Type Culture Collection

FOR ATCC USE ONLY

Signature: ____________________ **Printed Name:** ____________________

Date: ____________________ **Title:** ____________________

Customer Acceptance of Responsibility

On behalf of the Institution identified below, we acknowledge that the materials requested from American Type Culture Collection (ATCC) on behalf of BEI Resources represent a potential hazard to the public health and/or agricultural crops or products, including but not limited to viable microorganisms or toxins that may cause human disease. This Customer Acceptance of Responsibility (CAR) acknowledges the shipping address provided below meets all certifications on this form. Note: If multiple shipping addresses are needed, a CAR form must be submitted for each address.

PLEASE PRINT

Institution: ____________________

Department: ____________________

Shipping Address: ____________________

City: ____________________

State: ____________________ Postal code: ____________________

Country: ____________________

Telephone: ____________________ Fax: ____________________

On behalf of the Institution, we hereby certify that our Institution shall: (1) ensure that only qualified scientists work with these materials in proper facilities; (2) provide sufficient internal security to assure access to these materials only by those individuals authorized to work with them; (3) not transfer, export, resell, or otherwise dispose of any items supported by this statement to any third party under any circumstances without express written authorization from ATCC and the appropriate government agencies; (4) not permit access to the materials by foreign nationals when to do so would constitute a violation of export control laws; (5) maintain adequate insurance coverage for liability to any party that might be injured by release of these materials; (6) comply with all applicable federal, state, or local laws and regulations pertaining to these materials or their handling, storage, use, transportation; and (7) destroy all materials according to accepted practices for destruction of microbiological cultures or reagents upon completion of work.

We understand that by providing this signed form to ATCC on behalf of BEI Resources we are accepting responsibility for these agents and all risks associated with handling of them in our facility, as well as any adverse events resulting from our violation of the security requirements or unauthorized dissemination of the agents.

Print Full Name of Registrant: ____________________

Signature: ____________________ Date: ____________________

The undersigned officer of the Institution certifies that he or she has authority to make the above certifications and representations on behalf of the Institution and further warrants that he or she is legally authorized to enter into binding agreements on behalf of the Institution.

Print Full Name of Authorized Institutional Official: ____________________

Signature: ____________________ Date: ____________________

Title: ____________________
