# Supplementary material for: The NIH-NIAID Schistosomiasis Resource Center at the Biomedical Research Institute: Molecular Redux
Source: PLoS Negl Trop Dis. 2016 Oct 20;10(10):e0005022. doi: 10.1371/journal.pntd.0005022 (PMC5072641; doi:10.1371/journal.pntd.0005022)
Supplement: S2 File — The SRC at BRI offers a life cycle course twice yearly and an annual molecular skills course. Included are example outlines for each course. While the life cycle course features recurring but continually refined content, the molecular course topic is changed yearly to track updates in the field. (PDF) [file pntd.0005022.s002.pdf]

Schistosoma spp. Life Cycle Training Course

April 13-15, 2016

Schistosomiasis Resource Center
Biomedical Research Institute
12111 Parklawn Drive Rockville, MD 20852

Wednesday, April 13 (*Lectures held in Room 103, lab procedures in Room 213)*

9:00-9:15AM	**Meet and Greet**<br>SRC Staff<br>Life Cycle Course Attendees	Room 103
9:15-9:20AM	**Welcome to BRI**<br>Paul Nisson, PhD<br>Executive Director	Room 103
9:30-10:00AM	**Schistosomiasis pathology in the field and in the lab**<br>Michael Hsieh, MD, PhD<br>Principal Investigator, BRI	Room 103
10:00-10:30AM	***Biomphalaria glabrata* maintenance**<br>André Miller	Room 103
10:30-11:00AM	***Bulinus truncatus* and *Oncomelania hupensis* maintenance**<br>Sarah Li	Room 103
11:00-11:30AM	**Maintaining *S.mansoni* in the laboratory**<br>Kenia Villatoro-Benitez	Room 103
11:30-11:50AM	**Perfusions: collecting adult worms from *S.mansoni*-infected mice**<br>André Miller	Room 103
11:50-12:30PM	**LUNCH**	
12:30-1:00PM	**Tour of *B.glabrata, O. hupensis and B.truncatus colonies***<br>Kenia Villatoro Benitez<br>Sarah Li	Room 213 SRC
1:-3:30PM	**Perfusions of *S.mansoni*-infected mice to collect adult schistosomes**<br>André Miller<br>Kenia Villatoro Benitez	Room 213 SRC<br>Student participation
3:30PM	**DISMISSAL**	

Schistosoma spp. Life Cycle Training Course

Thursday, April 14

9:00-9:30AM	**NIH Grants and Research Resources**<br>Glen C. McGugan, Jr, PhD<br>Program Officer<br>Parasitology and International Programs Branch<br>National Institute of Allergy and Infectious Diseases, NIH<br>Rockville, MD	Room 103
9:30-10:00AM	**Maintaining *S.haematobium* in the laboratory: from snail to hamster and back again**<br>Taiwo Ogunbayo	Room 103
10:00-10:30AM	**Collecting *S. haematobium* cercariae from patent *Bulinus***<br>Sarah Li<br>Taiwo Ogunbayo	Room 213 SRC
10:30-11:00AM	**Collecting *S. japonicum* cercariae from patent *O.hupensis***<br>Sarah Li	Room 213 SRC
11:00--12:30PM	**Collecting *S.mansoni* cercariae from patent *B.glabrata***<br>Kenia Villatoro Benitez<br>André Miller	Room 213 SRC Lab<br>Student participation
12:30-1:30PM	**LUNCH**	
1:30-1:45PM	**Percutaneous exposure of hamsters for *S.haematobium* infection**<br>Demonstration<br>Taiwo Ogunbayo	Room 208 SRC
1:45-2:15PM	**Percutaneous exposure of mice for *S.mansoni* infection**<br>Demonstration<br>Kenia Villatoro Benitez	Room 208 SRC
2:15-3:00PM	**Perfusion of *S.haematobium*-infected hamster to collect adult worms**<br>Demonstration<br>Sarah Li and Taiwo Ogunbayo	Room 213 SRC
3:00-4:00PM	**Preparation of *in vitro* schistosomules of *S.mansoni***<br>Demonstration<br>André Miller	Room 213 SRC
4:00PM	**DISMISSAL**	

Schistosoma spp. Life Cycle Training Course

Friday, April 15

9:00-10:00AM	**Isolation of viable *S.mansoni* eggs from liver tissue**<br>Kenia Villatoro Benitez<br>André Miller	Room 213 SRC<br>Student participation
10:00-11:30AM	**Hatching *S.mansoni* eggs for miracidia collection**<br>**Exposing snails to miracidia**<br>Kenia Villatoro Benitez<br>André Miller	Room 213 SRC<br>Student participation
11:30-12:30PM	**LUNCH /DISMISSAL**<br>BRI staff available for questions	Room 103

BRI
BIOMEDICAL RESEARCH INSTITUTE

2015 SCHISTOSOMIASIS MOLECULAR TRAINING COURSE

Evaluation of DNA methylation of *Biomphalaria glabrata* in normal vs. thermal stress conditions

Schistosomiasis Resource Center
Biomedical Research Institute
Rockville, MD

July 13-15, 2015

MONDAY JULY 13

9:00-9:15 AM	Welcome to BRI	**Paul Nisson, PhD**<br>Executive Director<br>BRI
9:15-9:30 AM	Introduction to BRI	**Michael Hsieh, MD, PhD** / **MMK** overview of SRC<br>Principal Investigator, BRI
	to SRC	Welcome and introduction, BRI mission, SRC staff resources
9:30-10:30 AM	Lecture	**Michael Hsieh, MD, PhD** (BRI)<br>"Host DNA methylation induced by experimental urogenital Schistosomiasis"
10:30-10:45 AM	Break	
10:45-11:30 AM	Lecture	**Wannaporn Ittiprasert, PhD** (SRC)<br>"Current epigenetic study methods focusing on invertebrates"<br><br>Experiment workflow
11:30 AM-12.30 PM	Lunch	
12:30-2:45 PM	Lab	Dual DNA and RNA extraction (Ittiprasert and Miller)
2:45-3:00 PM	Break	
3:00-3:30 PM	Lab	DNA and RNA qualification and quantification (Ittiprasert and Miller)

TUESDAY JULY 14

9:00-10:15 AM	Lab	Determining percent quantification of DNA methylation<br>(Ittiprasert and Miller)
10:15-10:45 AM	Break	
10:45-11:15AM	Lecture	**John Satterlee, Ph.D.**<br>Program Coordinator, NIH Roadmap Epigenomics Program<br>National Institute on Drug Abuse, NIH
11:15-11:45 AM	Lecture	**Ankit Gupta, Ph.D.**<br>Laboratory of Malaria and Vector Research<br>National Institute of Allergy and Infectious Diseases, NIH<br>Rockville, MD<br>“Conditional knockdown suggests *P.falciparum* CLAG3 oligomers contribute to PSAC function”
11:45AM-12: 45PM	Lunch	
12:45-1:45 PM	Lecture	**Jeffrey Bethony, Ph.D.**<br>Department of Microbiology, Immunology and Tropical Medicine<br>George Washington University<br>Washington D.C.<br>“microRNA and gene regulation in helminthes”
1:30-2.45 P.M. Lab		Data analysis of methylation level (Ittiprasert and Miller)<br><br>Experiment for gene expression by real time PCR<br>(Ittiprasert and Miller)

WEDNESDAY JULY 15

9:00-9:30AM	Lecture	**Glen Mcgugan, Ph.D.**<br>Program Officer, Parasitology and International Programs<br>DMID/NIAID/NIH<br>Rockville, MD<br>“NIH Grants and Research Resources”
9:30-11:00 A.M.	Lab	Gene expression and correlation analysis between DNA methylation and gene expression in *B. glabrata* snail<br>(Ittiprasert and Miller)
		Conclusion
